# Can Resistance Training Prevent Breast Cancer-Related Lymphedema? A Systematic Review with Meta-Analysis

**DOI:** 10.3390/jcm15093297

**Published:** 2026-04-26

**Authors:** Raúl Alberto Aguilera-Eguía, Carlos Zaror, Ruvistay Gutiérrez-Arias, Olga Patricia López, Héctor Fuentes-Barria, Barbara Burgos Mansilla, Ángel Roco-Videla, Naira Figueiredo Deana, Mariana Melo-Lonconao, Xavier Bonfill, Pamela Serón

**Affiliations:** 1 Departamento de Salud Pública, Facultad de Medicina, Universidad Católica de la Santísima Concepción, Concepción 4090541, Chile; 2 Doctorado en Metodología de la Investigación Biomédica y Salud Pública, Universidad Autónoma de Barcelona, 08193 Barcelona, Spain; 3 Departamento de Odontopediatría y Ortodoncia, Facultad de Odontología, Universidad de La Frontera, Temuco 4811230, Chile; carlos.zaror@ufrontera.cl (C.Z.); naira.figueiredo@ufrontera.cl (N.F.D.); 4 Center for Research in Epidemiology, Economics and Oral Public Health (CIEESPO), Faculty of Dentistry, Universidad de La Frontera, Temuco 4811230, Chile; xbonfill@santpau.cat; 5 Centro de Excelencia CIGES, Universidad de La Frontera, Temuco 4811230, Chile; pamela.seron@ufrontera.cl; 6 Departamento de Apoyo en Rehabilitación Cardiopulmonar Integral, Instituto Nacional del Tórax, Santiago 7500921, Chile; ruvistay.gutierrez@gmail.com; 7 Exercise and Rehabilitation Sciences Laboratory, School of Physical Therapy, Faculty of Rehabilitation Sciences, Universidad Andres Bello, Santiago 7591538, Chile; 8 INTRehab Research Group, Instituto Nacional del Tórax, Santiago 8580745, Chile; 9 Departamento de Salud Oral, Facultad de Salud, Universidad Autónoma de Manizales, Pereira 660003, Colombia; sonrie@autonoma.edu.co; 10 Centro de Investigación en Medicina de Altura (CEIMA), Universidad Arturo Prat, Iquique 1110939, Chile; hefuentes_@unap.cl; 11 Departamento de Ciencias de la Rehabilitación, Facultad de Medicina, Universidad de La Frontera, Temuco 4811230, Chile; barbara.burgos@ufrontera.cl; 12 Dirección de Desarrollo y Postgrados, Universidad Autónoma de Chile, Santiago 7500912, Chile; angel.roco@uautonoma.cl; 13 Doctoral Program in Sciences in Applied Cellular and Molecular Biology, Universidad de La Frontera, Temuco 4811230, Chile; 14 Departamento Nacional de Salud Pública, Facultad de Medicina, Universidad San Sebastián, Sede Concepción 4081339, Chile; mariana.melo@uss.cl; 15 Clinical Epidemiology and Public Health, Biomedical Research Institut Sant Pau, 08025 Barcelona, Spain

**Keywords:** breast neoplasms, breast cancer lymphedema, early diagnosis, resistance training, weightlifting exercise program

## Abstract

**Introduction:** Breast cancer-related lymphedema (BCRL) affects quality of life (QoL) and increases healthcare costs. Resistance training (RT) is proposed as a preventive strategy, although its safety and effectiveness remain uncertain. **Objective:** To evaluate the effectiveness and safety of RT in preventing BCRL in women at risk. **Methods:** MEDLINE, Embase, CENTRAL, PEDro, and LILACS databases were searched from their inception to January 2025, along with the gray literature, trial registries, and preprints. Risk of bias was assessed using RoB 2, and certainty of the evidence (CoE) was assessed using GRADE. Primary outcomes were the occurrence of lymphedema and overall QoL; secondary outcomes included pain, upper limb function, range of motion (ROM), grip strength, and adverse events. **Results:** Eight RCTs (*n* = 1131) were included. The effects of RT on lymphedema and arm volume are very uncertain (very low CoE). For QoL, pain, ROM, and grip strength, the findings were inconsistent and uncertain (low to very low CoE). Adverse events were mild and transient, with no serious complications. **Conclusion:** RT is probably safe in women at risk of developing BCRL. Its preventive effectiveness is highly uncertain. Well-designed RCTs with standardized diagnostic criteria, patient-centered outcomes, and long-term follow-up are needed to establish their role in BCRL prevention with greater certainty. **Ethics and dissemination:** This study did not require ethical approval. The results will be disseminated through publications in peer-reviewed journals and academic presentations. **Registration:** PROSPERO (CRD42023455720).

## 1. Introduction

Breast cancer is the most common neoplasm in women, with approximately 2.5 million new cases in 2022 [1]. Although mortality has declined in high-income countries, in transitioning regions such as South America and Asia, both incidence and mortality continue to rise [1]. This situation increases the number of women undergoing surgery for breast cancer who are at risk of developing chronic complications, including breast cancer-related lymphedema (BCRL). Importantly, this risk is not uniform across all women but rather spans a heterogeneous spectrum depending on patient- and treatment-related factors [2,3]. BCRL is a chronic, progressive condition resulting from damage to the lymphatic system caused by cancer treatments [4,5]. It is characterized by the accumulation of protein-rich interstitial fluid in subcutaneous tissues, which increases limb volume and is associated with stiffness, pain, and functional limitation [6,7]. In advanced stages, it can cause fibrosis and irreversible trophic changes, affecting quality of life (QoL) in its physical, emotional, and social dimensions, and increasing the risk of recurrent infections [8,9]. Its prevalence ranges from 3% to 75% [2,10], with a higher risk after extensive axillary dissection and radiotherapy, especially during the first two years, although the danger persists for life [2,10]. Risk factors include the extent of lymph node dissection, radiotherapy, the use of taxanes, obesity, previous infections in the arm, and smoking [11,12]. In addition to causing recurrent complications, BCRL represents a considerable economic burden, reinforcing the need for effective prevention and early detection strategies [13].

One of the main therapeutic interventions is physiotherapy, aimed at reducing the limb volume through specialized techniques and compression measures [2,9,14]. However, this approach entails a high cost for both patients and health systems [15]. For decades, clinical guidelines discouraged exercise in women with BCRL or at risk, based on the assumption that mechanical stress could aggravate edema [16,17]. However, recent evidence from systematic reviews (SRs) indicates that supervised and progressive resistance training (RT) is safe and can improve symptoms and function in women with BCRL [18,19]. Furthermore, in the preventive field, RT could reduce the risk of BCRL by promoting lymphatic return, maintaining joint mobility, strengthening shoulder muscles, modulating inflammation, and stimulating vascular and lymphatic repair [20,21]. However, evidence of its preventive effectiveness remains limited and heterogeneous. In particular, existing studies differ in key aspects, including resistance training protocols (such as intensity, frequency, progression, supervision, and intervention duration), the diagnostic criteria used to identify breast cancer-related lymphedema (BCRL), and follow-up periods. Despite this, international organizations such as the Academy of Oncologic Physical Therapy of the American Physical Therapy Association (APTA) and the American College of Sports Medicine (ACSM) recommend supervised, progressive RT as a preventive strategy for women at risk [22,23,24,25,26,27,28]. To date, only one SR has been identified that evaluated conservative interventions for the prevention of BCRL, including RT. However, it was limited to studies published up to 2013. It did not incorporate subgroup or sensitivity analyses due to the small number of randomized clinical trials (RCTs) available at that time [21]. Since then, the evidence base has expanded, enabling an updated and more methodologically robust synthesis of the preventive role of RT in BCRL, including subgroup and sensitivity analyses that were not feasible in the previous review. In this context, the present SR aims to analyze its safety and effectiveness in women at risk, considering its short-, medium-, and long-term impact on clinically relevant outcomes.

## 2. Materials and Methods

### 2.1. Protocol and Registration

This SR was conducted following the guidelines established in the Cochrane Handbook for Systematic Reviews of Interventions [29] and is reported in accordance with the PRISMA (Preferred Reporting Items for Systematic Reviews and Meta-Analyses) checklist [30] (see Supplementary Materials S1 and S2). The protocol was previously registered in PROSPERO (CRD42023455720) and published [31].

### 2.2. Selection Criteria

#### Type of Studies

Only RCTs that evaluated the effectiveness of RT in women at risk of developing BCRL were included. Only studies published in English, Spanish, or Portuguese were considered.

### 2.3. Type of Participants or Populations of Interest

Studies were included that evaluated women undergoing surgical treatment for breast cancer, which left them at risk of developing BCRL. This included surgical procedures such as axillary lymph node dissection, sentinel lymph node biopsy, axillary clearance, or radiotherapy targeting the axilla and supraclavicular fossa.

Studies including participants with previously diagnosed BCRL or recurrent breast cancer were generally excluded. Nevertheless, if only a small proportion of participants did not meet the eligibility criteria, the study could still be retained provided that data for eligible participants were reported separately or that eligible participants accounted for at least 80% of the total sample, in order to avoid unnecessary loss of relevant information [32].

When detailed information on the proportion of eligible participants was not available, attempts were made to contact the corresponding authors to obtain the required data. If no response was received or if the percentage of eligible participants was less than 80%, the study was excluded [32].

### 2.4. Type of Interventions

Interventions that included RT aimed at muscle strengthening, using weights, elastic bands, or body weight, were considered eligible. The studies had to specifically evaluate the preventive effects of RT on BCRL. Studies analyzing combined interventions within physical therapy programs were also included, provided that the comparison group received the same interventions without the RT component, in order to isolate the specific effect of RT.

### 2.5. Comparators

The control groups consisted of participants who received patient education only, did not perform any exercise intervention, or participated in exercise modalities other than RT, such as aerobic exercises or programs focused on improving flexibility.

### 2.6. Type of Outcomes

#### 2.6.1. Primary Outcomes

- Occurrence of lymphedema: Considered as a dichotomous outcome (presence or absence of the event) or continuous outcome (volume or percentage change in the volume of the affected arm), according to the reports of the included studies. Only studies that used objective and predefined diagnostic criteria to determine the presence of lymphedema were accepted. Accepted methods included circumference measurement, water displacement, electrical bioimpedance, laser scanning, perimetry, or dual-energy X-ray absorptiometry (DEXA). Studies based solely on clinical judgment, without objective measurements or participants’ self-reports, were excluded.
- Quality of life (QoL): Assessed using validated self-administered instruments, both generic and specific, such as the EORTC QLQ-C30 questionnaire or other internationally recognized scales.

#### 2.6.2. Secondary Outcomes

- Pain intensity: Measured using validated scales, such as the Visual Analog Scale (VAS).
- Upper limb function: Assessed using tools such as the Disabilities of the Arm, Shoulder, and Hand (DASH) questionnaire.
- Range of motion (ROM): Determined using goniometry or other validated measuring instruments.
- Grip strength: Assessed using dynamometry.
- Adverse events: Defined as an increase in lymphedema volume, the onset of pain related to the intervention, etc.

### 2.7. Duration of Follow-Up

Follow-up periods were classified into three categories: short term (≤3 weeks), medium term (>3 weeks to 6 weeks), and long term (≥6 weeks).

### 2.8. Information and Search Sources

#### Search Strategy

A comprehensive search for RCTs was conducted in the Medline/PubMed, Embase, CENTRAL, PEDro, and LILACS databases, from their inception to January 2025. Specific details of the search strategies for each database are presented in Supplementary Materials S3. To ensure comprehensiveness and minimize publication bias, additional sources were considered, including the gray literature (OpenGrey, Open Access Theses and Dissertations—OATD). The search also included clinical trial registries such as ClinicalTrials.gov, the WHO International Clinical Trials Registry Platform (ICTRP), and the Latin American and Caribbean Clinical Trials Registry. Preprint servers such as medRxiv and bioRxiv were also reviewed, and the reference lists of the included RCTs and relevant SRs were examined to identify additional potentially eligible studies.

### 2.9. Data Collection and Analysis

#### Selection of Studies

All results were exported to Rayyan software (web-based software; Rayyan Systems Inc., Cambridge, MA, USA) [33] (https://www.rayyan.ai/ (accessed on 15 January 2025)). After removing duplicate records, two researchers independently reviewed the titles and abstracts. They then independently assessed the full text of potentially eligible articles. Discrepancies were resolved by consensus among the reviewers, and reasons for study exclusion were documented in Supplementary Materials S4 and S5.

### 2.10. Data Extraction and Management

Four authors independently extracted data using a standardized, pre-tested form. From each included study, information was collected on study characteristics, baseline characteristics of participants, characteristics of the intervention and comparison groups, and the outcomes assessed. Discrepancies among reviewers were resolved by consensus.

### 2.11. Assessment of Risk of Bias

Four authors independently and in pairs assessed the risk of bias in each included study using the RoB 2.0 tool [34]. Discrepancies were resolved by consensus and, when this was not possible, the final decision was made by a third author. The RoB 2.0 tool assesses five domains: (1) bias arising from the randomization process, (2) bias due to deviations from planned interventions, (3) bias due to incomplete outcome data, (4) bias in outcome measurement, and (5) bias in the selection of reported outcomes [34]. Each domain contains guiding questions and allows for three possible judgments: low risk, some concerns, or high risk [34]. A graphic summary was developed to visually represent the results.

### 2.12. Addressing Missing Data

Although the protocol for this SR included strategies for handling missing data, such as contacting the authors of primary studies or performing statistical imputations (worst-case scenario method using STATA’s “metamiss” module), it was not necessary to implement them [29]. For the outcomes included in this review, the published reports provided sufficient data for extraction and synthesis, and therefore, no additional imputation procedures or author contact were required.

### 2.13. Estimation of the Treatment Effect

For dichotomous outcomes, such as the occurrence of lymphedema, relative risks (RRs) were calculated with their respective 95% confidence intervals (CIs). For continuous outcomes, such as arm volume, overall QoL, ROM, pain, and grip strength, mean differences (MDs) with 95% CI were used. When studies used different scales or units of measurement for the same outcome, standardized mean differences (SMDs) were calculated to allow comparison between studies. In cases where trials did not report sufficient data to calculate a point estimate of the effect or its corresponding CI, outcomes were presented descriptively, indicating the direction of the effect and, where possible, the certainty of the evidence.

### 2.14. Assessment of Heterogeneity

The heterogeneity among the included studies was assessed using the inconsistency statistic (I^2^), which quantifies the proportion of observed variability attributable to real differences between studies rather than random variation [35]. No fixed thresholds were applied for the interpretation of I^2^, given that this statistic can present high uncertainty, especially in meta-analyses with few studies. This information was considered in the interpretation of the results and incorporated into the assessment of the certainty of the evidence (CoE) according to the GRADE approach [36].

### 2.15. Assessment of Publication Biases

Publication bias was not assessed, as no meta-analysis in this SR met the minimum threshold of ≥10 studies required for reliable interpretation of funnel plots and for the application of statistical tests such as Begg’s or Egger’s [37]. This threshold is recommended to avoid unreliable results due to insufficient statistical power in publication bias analyses.

### 2.16. Data Synthesis

The selection of studies was presented using a PRISMA flow diagram [30], and the reasons for exclusion during the full-text assessment phase were detailed in a table. The characteristics of the included RCTs were summarized in structured tables. For each comparison, data were grouped based on similarities in participants, interventions, and outcomes and analyzed using a frequentist random effects model, employing the restricted maximum likelihood (REML) estimator, recommended for its robustness in handling heterogeneity across studies [38]. This model enabled accurate estimation of the variance between studies (τ^2^), thereby reducing bias in contexts with few studies. In studies with multiple relevant intervention arms, comparable groups were pooled to avoid data duplication and ensure a valid analysis. The results were presented in forest plots with 95% confidence intervals, following standard practices for quantitative synthesis in SRs [39].

All statistical analyses were performed using Stata 18 software (StataCorp, College Station, TX, USA). In cases where meta-analysis was not possible due to significant data gaps or considerable clinical heterogeneity, a structured narrative synthesis of the findings was performed, with attention to consistency across studies and their clinical relevance.

### 2.17. Subgroup Analysis and Investigation of Heterogeneity

Although subgroup analyses were planned by RT type (intensity and frequency) and by the scale used to assess QoL, they could not be performed because the total number of included studies was less than 10, which would have compromised methodological validity and statistical power [40]. In addition, clinical heterogeneity across studies in terms of intervention protocols, comparator groups, diagnostic criteria for lymphedema, and follow-up duration further limited the feasibility and interpretability of subgroup analyses.

### 2.18. Sensitivity Analysis

Three sensitivity analyses were performed to assess the robustness of the results [40]:

1. For overall QoL (>6 weeks), we compared SMD versus MD because the included studies used different validated instruments to assess this outcome, and this analysis allowed us to examine whether the findings were sensitive to the choice of effect measure.
2. We assessed the influence of lymphedema diagnostic criteria (≥10%, ≥5–10% excluding >3%, and all criteria combined) because the included studies applied different thresholds to define lymphedema, which could contribute to heterogeneity in the pooled estimates.
3. We excluded studies with a high risk of bias to explore the stability of the findings after removing methodologically weaker evidence. In addition, because one of these studies included a very small proportion of participants with baseline lymphedema (<2%), this analysis also allowed us to explore the potential influence of that trial on the overall direction of the findings.

### 2.19. Summary of Findings Table

The certainty of the evidence (CoE) was assessed using the GRADE (Grading of Recommendations, Assessment, Development and Evaluation) approach, which considers five domains: risk of bias, inconsistency, indirectness, imprecision, and publication bias [41]. According to this approach, CoE is classified into four levels: high, moderate, low, or very low [42]. The results were interpreted using a minimally contextualized approach, which prioritizes the CI limit closest to the absence of effect to determine whether the estimate suggests significant benefit or harm. The assessment was performed using GRADEpro GDT software (https://gdt.gradepro.org/app/ (accessed on 19 June 2025)), and a Summary of Findings Table was generated for each outcome of interest. These tables are presented in the Supplementary Materials.

### 2.20. Use of Generative AI

During the preparation of this manuscript, the authors used ChatGPT (OpenAI, GPT-5.4 Thinking) exclusively for language editing, specifically to improve wording, grammar, and overall readability. The tool was not used for study selection, data extraction, data analysis, risk-of-bias assessment, interpretation of results, or the formulation of scientific conclusions. After using this tool, the authors critically reviewed and edited the content as needed and took full responsibility for the final content of the manuscript.

## 3. Results

### 3.1. Study Selection

**Figure 1:**
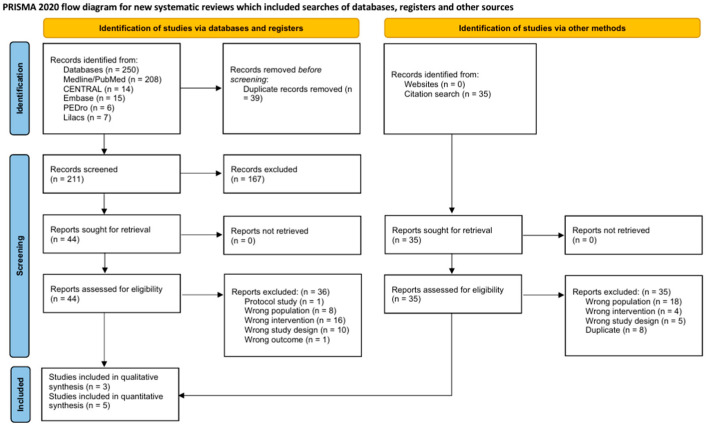
PRISMA 2020 flow diagram.

A total of 250 records were identified through the databases consulted. After removing 39 duplicates, two reviewers independently evaluated 211 titles and abstracts, excluding 167 that did not meet the eligibility criteria. Subsequently, 44 full-text articles were evaluated [24,26,43,44,45,46,47,48,49,50,51,52,53,54,55,56,57,58,59,60,61,62,63,64,65,66,67,68,69,70,71,72,73,74,75,76,77,78,79,80,81,82,83,84], of which 8 studies met the inclusion criteria [24,26,43,44,45,46,47,48], In addition, the reference lists of previous SRs were examined [85,86,87,88,89,90,91,92,93,94,95,96,97,98,99,100,101,102,103,104,105,106,107], but no additional eligible studies were identified. No ongoing trials meeting the criteria for this SR were found either. The reasons for exclusion of the studies evaluated in full text are detailed in Supplementary Materials S4 and S5. Figure 1 presents the PRISMA flow diagram corresponding to the selection process.

### 3.2. Characteristics of the Included Studies

Eight RCTs were included [24,26,43,44,45,46,47,48], with a total of 1131 women undergoing surgical treatment for breast cancer and at risk of developing BCRL. The sample size ranged from 60 [45] to 242 [24] participants. All studies used a parallel group design with individual randomization. Two studies were conducted in the United States [26,44], and the remaining studies were conducted in Denmark [43], Spain [45], Norway [48], Canada [24], Australia [46], and Germany [47]. The mean age of participants ranged from 49.2 to 61.7 years. All studies recruited women exclusively. Regarding cancer stage, six studies included women with stages I to III, while two studies [45,47] did not specifically report this information. In terms of cancer treatment, most participants had received chemotherapy, hormone therapy, and/or radiotherapy. The types of surgery included mastectomy, lumpectomy, axillary dissection, sentinel node biopsy, and breast-conserving surgery. Although all studies focused on women at risk of developing BCRL [24,26,43,44,45,47,48], one study included a small number of participants with a previous diagnosis of lymphedema [46]. This study was considered eligible because, in the intervention group, only one participant (1% of 104) had a baseline circumference difference > 2 cm. In contrast, in the control group, two participants (2% of 100) were reported to have the same condition and met the established inclusion criteria. Regarding ethnic distribution, two studies reported specific data. In one study, 89% of participants were Caucasian, and 12% were African American [44]; in the other, 70% were white, 24% were non-white, and 6% belonged to other ethnic groups [26]. The other studies did not report this information. The general methodological and clinical characteristics of the included studies are detailed in Table 1.

### 3.3. Interventions and Comparisons

The RT interventions evaluated in the included studies were heterogeneous in terms of duration, frequency, intensity, and level of supervision. In general, the programs consisted of progressive RT focused on strengthening the upper and lower extremity muscles. The duration of the interventions ranged from 6 weeks to 18 months, with a frequency of 1 to 3 sessions per week. Training intensity was adjusted using different criteria. Four studies used the percentage of one rep max (1RM) to define and progress the workload [24,43,44,45], with the latter using 7RM adjusted monthly according to fatigue [44]. Three studies [26,47,48] reported gradual or tolerance-based progression, but did not specify the exact method of load adjustment. One study [46] did not provide information on the criteria used to adjust training intensity. Regarding the level of supervision, four studies implemented full supervision, in which all sessions were led by health professionals [24,45,47,48]. Four studies used a partial supervision scheme, which combined supervised sessions with self-administered components [26,43,44,46]. No study used a completely unsupervised program.

The comparison groups were classified into three categories, according to the nature of the control group intervention [41]:

1. Supervised physical activity restriction [48].
2. Usual care or absence of structured exercise [26,43,44,45,46].
3. Structured aerobic exercise [24].

In one of the included studies [24], which incorporated three parallel arms (RT, aerobic exercise, and a control group), each comparison was analyzed separately to avoid data duplication. The detailed characteristics of the interventions and their respective comparator groups are summarized in Table 2. This classification of comparators was performed to improve clinical consistency and comparability of the analyses, in accordance with the methodological recommendations of the Cochrane Handbook for Systematic Reviews of Interventions [41].

### 3.4. Outcomes

#### 3.4.1. Primary Outcome: Occurrence of Lymphedema

Lymphedema occurrence was the primary outcome in the included studies. It was assessed using different methods, including water displacement and circumference measurements, and applying various diagnostic cut-off points. One study defined it as a ≥10% increase in inter-arm volume measured by water displacement [48]; another applied a threshold >3% [43]; a third used a difference ≥10% calculated from circumference measurements [46]; and another defined lymphedema as an absolute difference ≥200 mL between both arms [24]. In an additional study, volume was measured by water displacement as a continuous outcome, without applying a diagnostic cut-off point [44]. The specific characteristics of the measurement methods, formulas used, and diagnostic criteria are summarized in Table 3.

#### 3.4.2. Primary Outcome: Overall Quality of Life (QoL)

Three of the included studies assessed overall QoL using validated instruments [44,45,47]. Two of them used the FACT-B (Functional Assessment of Cancer Therapy-Breast) questionnaire, designed specifically for breast cancer patients [44,45]. The third study used the EORTC QLQ-C30 instrument, a generic questionnaire for cancer patients, focusing the analysis on the overall health status domain [47]. In all three studies, total scores were considered the primary outcome for measuring overall QoL. The characteristics of the instruments used and the specific dimensions assessed are detailed in Table 4.

#### 3.4.3. Secondary Outcome

Five of the included studies evaluated clinically relevant secondary outcomes [48]. Pain intensity was measured in one study using the Visual Analog Scale (VAS) [48]. Shoulder ROM was evaluated in three studies [43,45,46], using different instruments (digital inclinometer, manual goniometer, and electrogoniometer). Manual grip strength was assessed in one study [45], using dynamometry as a measurement tool. Six studies reported adverse events [24,26,43,44,45,48]. Three of them used structured clinical reports [24,44,48], one implemented a safety log during sessions [26], another used self-reporting supplemented with clinical monitoring [43], and one used a supervised log throughout the intervention [45]. The most frequently reported adverse events included muscle discomfort, joint overload, shoulder pain, dizziness, and transient increases in arm volume. In all cases, the events were mild and did not interfere with the continuity of the exercise programs. None of the studies included assessed overall upper limb function using standardized tools or equivalent instruments. The methods and instruments used for each outcome are presented in detail in Table 5.

### 3.5. Risk of Bias in the Included Studies

Of the eight RCTs included, one was classified as having a high risk of bias [46], six raised some concerns [24,26,43,44,47,48], and one was considered to have a low overall risk [45]. The domain that most frequently presented limitations was D1 (bias arising from the randomization process), in which five studies did not provide sufficient information on the generation or concealment of the allocation sequence. In D2 (bias due to deviations from intended intervention), six studies were classified as having “some concerns” due to the absence of participant and staff blinding, as well as the use of per-protocol analysis rather than intention-to-treat analysis. In one study [46], these limitations were more pronounced, and it was therefore classified as high risk. Domain D3 (bias due to missing outcome data) raised some concerns across several studies, especially when losses to follow-up were not addressed through imputation or sensitivity analyses. In D4 (bias in measurement of the outcome), most trials were considered to be at low risk, although one study was classified as high risk when evaluating subjective outcomes without blinding [46]. In D5 (bias in selection of the reported result), all trials were assessed as low risk because the stated outcomes were consistently reported (see Supplementary Materials S6).

### 3.6. Effects of Interventions

Upper limb function was pre-specified as an outcome of interest; however, it was not evaluated in any of the included studies and therefore could not be analyzed in this review.

#### 3.6.1. Comparison 1 Resistance Training (RT) Versus Activity Restriction

This comparison was based on a single study [48] with 204 participants.

- Occurrence of lymphedema (>6 weeks): The effect of RT on the occurrence of lymphedema remains uncertain (RR = 1.04; 95% CI: 0.51 to 2.09; one RCT [48], 204 participants, very low CoE) (see Supplementary Materials S7).
- Arm volume (>6 weeks): The effect of RT on arm volume remains uncertain (MD = −30.00; 95% CI: −73.64 to 13.64; one RCT [48], 204 participants, very low CoE) (see Supplementary Materials S7).
- Pain (>6 weeks): Evidence is inconclusive regarding the effect of RT on pain (one RCT [48], 204 participants, very low CoE). The available data were reported descriptively and suggested that RT could increase pain at 6 months, although no differences were observed at 24 months (see Supplementary Materials S7).
- Adverse events: One RCT [48] with 204 participants reported three musculoskeletal events in the RT group: two cases of adhesive capsulitis (one possibly pre-existing) and one case of supraspinatus tendinopathy. No adverse events were reported in the comparison group.

#### 3.6.2. Comparison 2 Resistance Training (RT) Versus Usual Care/No Structured Exercise

This comparison was evaluated across five studies [24,26,43,44,46] involving a total of 534 participants.

- Occurrence of lymphedema (>3 weeks to 6 weeks): The effect of RT on the occurrence of lymphedema remains uncertain (RR = 0.50; 95% CI: 0.13 to 1.93; one RCT [24], 164 participants, very low CoE) (see Supplementary Materials S8).
- Occurrence of lymphedema (>6 weeks): The effect of RT on the occurrence of lymphedema remains uncertain (RR = 0.92; 95% CI: 0.53 to 1.61; three RCTs [26,43,46], 452 participants, very low CoE) (see Supplementary Materials S8).
- Arm volume (>6 weeks): Evidence is inconclusive regarding the effect of RT on arm volume (one RCT [44], 82 participants, very low CoE). The RT group showed a smaller increase in arm volume than the usual care group (+27.3 mL vs. +57.4 mL), but the difference was not statistically significant (*p* = 0.535) (see Supplementary Materials S8).
- Overall quality of life (>6 weeks): The effect of RT on overall quality of life remains uncertain (SMD = 0.25; 95% CI: −0.60 to 1.09; two RCTs [44,47], 131 participants, very low CoE). Descriptive findings from one additional RCT [46] with 60 participants suggested that RT may not have a significant effect (MD = −2.9; 95% CI: −7.0 to 2.1; moderate CoE) (see Supplementary Materials S8).

##### Secondary Outcomes

- Shoulder flexion (>6 weeks): Three RCTs [43,45,46] with 331 participants evaluated this outcome. RT may not have a significant effect on shoulder flexion (MD = −1.00; 95% CI: −5.64 to 3.64; one RCT [43], 130 participants, low CoE). Descriptive findings from two additional RCTs also suggested no significant effect (MD = 1.9; 95% CI: −4.5 to 8.2; RCT [45], 141 participants, very low CoE; MD = −2.0; 95% CI: −8.3 to 4.4; RCT [47], 60 participants, low CoE) (see Supplementary Materials S8).
- Shoulder abduction (>6 weeks): Two RCTs [43,46] with 271 participants evaluated this outcome. RT may not have a significant effect on shoulder abduction (MD = −2.00; 95% CI: −11.86 to 7.86; one RCT [43], 130 participants, very low CoE). Descriptive findings from one additional RCT [46] with 141 participants suggested a greater increase in shoulder abduction in the RT group (MD = 10.0; 95% CI: 3.6 to 16.5; very low CoE) (see Supplementary Materials S8).
- External shoulder rotation (>6 weeks): Two RCTs [43,46] with 281 participants evaluated this outcome. The evidence is very uncertain about the effect of RT on external shoulder rotation (MD = 1.00; 95% CI: −5.02 to 7.02; one RCT [43], 130 participants; very low CoE). Descriptive findings from one additional RCT [46] with 151 participants also suggested no clear differences between groups (MD = −1.2; 95% CI: −6.2 to 3.8; very low CoE) (see Supplementary Materials S8).
- Pain (>6 weeks): RT may not have a significant effect on pain (one RCT [45], 60 participants, moderate CoE). Descriptive findings indicated no significant differences between groups, with a similar distribution of pain scores; however, the absence of point estimates and confidence intervals limits interpretation (see Supplementary Materials S8).
- Grip strength (>6 weeks): RT may not have a clinically relevant effect on grip strength (MD = 0.2; 95% CI: −1.3 to 1.6; one RCT [45], 60 participants, low CoE) (see Supplementary Materials S8).
- Adverse events: Six studies reported adverse events with RT [24,26,43,44,45,48]. In general, the events were mild and did not affect adherence. Cases of lymphedema, muscle pain, overload, dizziness, and shoulder discomfort were reported. Some events were considered possibly related to the intervention. In one study, increases in arm volume were observed, although they did not persist in the long term.

#### 3.6.3. Comparison 3 RT vs. Aerobic Training

This comparison was evaluated in a study [24] with 160 participants.

- Occurrence of lymphedema (>3 to 6 weeks): The effect of RT versus aerobic training on the occurrence of lymphedema remains uncertain (RR = 0.41; 95% CI: 0.11 to 1.52; one RCT [24], 160 participants, very low CoE) (see Supplementary Materials S9).
- Adverse events: A study with 44 participants [24] reported two adverse events possibly related to the treadmill stress test. Symptoms included dizziness, hypotension, nausea, and mild diarrhea. Both events were transient and resolved quickly without relevant clinical consequences.

### 3.7. Additional Analyses

The robustness of the RT results was explored using three sensitivity analyses:

- Change in effect measure (SMD vs. MD): The use of MD instead of SMD for the overall QoL outcome (>6 weeks) showed a possible improvement with RT (MD: 1.34; 95% CI: 0.28 to 2.39; very low CoE) (see Supplementary Materials S10 and S11).
- Diagnostic criteria for lymphedema: We evaluated whether the diagnostic definition influenced the effect of RT, considering three scenarios based on different inter-arm volume thresholds (≥10%, ≥5–10%, and >3%). The estimates were consistent with no changes in the direction of the effect. In all cases, the CoE was very low due to imprecision (see Supplementary Materials S12 and S13).
- Exclusion of studies with high risk of bias: Excluding such studies did not change the results. For the occurrence of lymphedema (>6 weeks), uncertainty regarding the effect of RT remained after excluding studies at high risk of bias (two RCTs, n = 305; low CoE).
- In secondary outcomes related to ROM, no clinically relevant effects were observed (see Supplementary Materials S14 and S15). Because the only study rated as having a high risk of bias also included a very small proportion of participants with baseline lymphedema (<2%), this analysis allowed us to explore the potential influence of that trial on the preventive focus and the overall direction of the findings.
- Adverse events: Five studies (24, 26, 43–45) reported RT-associated events, most of which were mild and had no impact on adherence. Cases of lymphedema, muscle pain, overload, dizziness, and shoulder discomfort were reported.

Additional analyses did not significantly alter the direction or magnitude of the RT effect. Although a possible improvement in overall QoL was observed, the CoE was very low. Consistency across diagnostic scenarios and the exclusion of studies with a high risk of bias reinforce the robustness of the results, despite limitations due to imprecision. The low frequency and severity of adverse events suggest that RT may be a safe intervention in this context.

## 4. Discussion

### 4.1. Summary of Key Findings

This SR identified potential effects of RT in women at risk of developing BCRL, although low and very low CoE limits confidence in the results.

In the comparison between RT and activity restriction, the evidence was very uncertain regarding the occurrence of lymphedema, arm volume, and pain (≥6 weeks). The adverse events reported were mild and short-lived.

Compared with usual care, the evidence on the occurrence of lymphedema was very uncertain in the short term (>3 to 6 weeks) and in the long term (>6 weeks). The evidence on arm volume (>6 weeks) was also very uncertain. Regarding overall QoL (>6 weeks), the findings were inconsistent. In some cases, the evidence was very uncertain, while in others, there may not have been a clinically relevant effect (moderate CoE). Although the results were interpreted using a minimally contextualized approach, the clinical significance of these QoL findings should be interpreted with caution and may be further informed by the minimal clinically important difference (MCID), when validated thresholds are available for the instruments used. In secondary outcomes, RT may not have a clinically relevant effect on shoulder flexion (>6 weeks) (low and very low CoE), while evidence on abduction (>6 weeks) and external rotation (>6 weeks) was very uncertain. For pain (>6 weeks), no significant differences were observed between groups (moderate CoE). Regarding grip strength (>6 weeks), a small increase was reported with RT, although the evidence was uncertain (low CoE). The adverse events reported were mostly mild and transient, with no impact on adherence.

Compared with aerobic exercise, the evidence on the occurrence of lymphedema (>3 to 6 weeks) is very uncertain. Regarding adverse events, two cases possibly related to aerobic exercise testing were reported; both were mild and transient, with no clinical repercussions.

These findings should be interpreted in light of previous evidence and current clinical guidance. The previous systematic review by Stuiver et al. [21] reported that progressive resistance training did not increase the risk of lymphoedema when symptoms were closely monitored and treated promptly if they occurred, although the authors emphasized the limited quality and heterogeneity of the available evidence. Similarly, current recommendations from the ACSM and the Academy of Oncologic Physical Therapy of APTA support progressive resistance training in women with or at risk of breast cancer-related lymphedema, particularly when introduced gradually and under professional supervision [22,23]. However, these recommendations are mainly grounded in evidence of safety and non-exacerbation rather than in high-certainty evidence demonstrating a preventive effect. In this context, our findings are broadly consistent with previous evidence and current guidance: RT appears to be safe when implemented cautiously, but uncertainty remains regarding its preventive effectiveness.

The substantial heterogeneity in diagnostic criteria for BCRL, RT protocols, and comparator interventions should also be considered when interpreting the pooled estimates. Differences in how lymphedema was defined and measured across trials may have introduced variability in outcome ascertainment, while variation in training frequency, intensity, progression, duration, and supervision likely limited the clinical comparability of the interventions being combined. In addition, the use of different comparators, such as usual care, activity restriction, or aerobic exercise, may have influenced both the magnitude and direction of the observed effects. Therefore, the pooled estimates should be interpreted with caution, as they may reflect an average effect across clinically and methodologically diverse studies rather than the effect of a single, uniform intervention.

### 4.2. Clinical Implications

This SR suggests that progressive resistance training (RT) could be a safe intervention in women at risk of developing BCRL. However, the available evidence is very uncertain regarding its effects on lymphedema occurrence, lymphedema volume, overall QoL, pain, grip strength, and ROM. This cautious clinical interpretation is broadly consistent with current guidance from the ACSM and APTA, which supports progressive resistance training primarily from a safety perspective. At the same time, the certainty of evidence regarding its preventive effectiveness remains limited. In clinical practice, RT could be implemented with caution within structured and individualized programs, ideally from the early postoperative stages [22]. Given the clinical complexity of this population, professional supervision may be considered a prudent measure to promote safety, adherence, and the monitoring of early signs of complications, rather than a recommendation directly derived from this review’s findings. RT could form part of a multimodal preventive approach, including prospective monitoring, compression measures in high-risk patients, self-care education, and periodic reassessments to detect early signs of lymphedema and adjust physiotherapy interventions in a timely manner. In this context, these programs may benefit from the involvement of professionals with training and experience in oncological rehabilitation, especially in clinical settings that allow continuous follow-up. The integration of RT into a continuous care model could facilitate early intervention when initial signs of lymphedema appear, potentially improving patients’ functionality and QoL. Currently, there are no cost-effectiveness studies on this intervention, which discourages its routine implementation in all contexts. In addition, logistical barriers such as time availability, transportation, or lack of motivation may affect adherence. In this regard, telemedicine and hybrid models (in-person and remote) could represent a promising alternative to improve access and continuity of programs, especially in resource-limited settings. Finally, the implementation of RT must consider health equity criteria and promote sustainable strategies that are culturally adapted to diverse populations. Patient participation in the prevention and self-care process should be promoted through empathetic, person-centered communication. Under these conditions, progressive RT could be implemented with caution within individualized programs while new high-quality evidence, with short-, medium-, and long-term follow-up, confirms its preventive effectiveness.

### 4.3. Implications for Future Research

This SR highlights significant gaps in research on RT as a preventive strategy for BCRL. Although supervised RT could be considered safe, the low or very low CoE limits its clinical applicability and highlights the need for RCTs with greater methodological rigor. Frequent methodological deficiencies were identified, including insufficient sample sizes, lack of blinding, variability in the diagnostic criteria for lymphedema, and considerable heterogeneity in the intervention protocols. These factors compromise the robustness of the conclusions and reinforce the need to standardize both interventions and study designs in future studies. It is recommended that RCTs be developed following recognized methodological frameworks, such as the SPIRIT guidelines for protocol development and CONSORT for RCT reporting. Furthermore, the heterogeneity of RT programs and the methods used to diagnose BCRL compromised comparability across studies and consistency in outcome measurement. Therefore, it is essential to establish clearly defined intervention protocols and adopt standardized diagnostic criteria, using validated and previously specified instruments. Another key gap in the available literature is the absence of standardized functional outcomes. Although some studies assessed specific outcomes, such as range of motion or grip strength, none evaluated overall upper limb function using standardized instruments. This limits the clinical interpretability of the findings and reduces comparability across studies. Future RCTs should incorporate standardized, valid, and clinically relevant functional outcomes to better capture the impact of RT on function in activities of daily living and patient-centered recovery. Another relevant limitation was the poor representation of diverse populations. Most studies were conducted in high-income countries and involved predominantly white participants, thereby limiting external validity and restricting the generalizability of the findings. It is considered necessary to promote multicenter RCTs that include multiethnic populations and those from underserved regions, in order to improve health equity and increase the clinical applicability of the results.

The lack of studies evaluating the feasibility and cost-effectiveness of RT also limits its implementation in real-world settings. Therefore, it is recommended that future research include economic evaluations and implementation studies that account for variables such as adherence, accessibility, and patient preferences across different settings. In this context, hybrid intervention models (in-person and remote) and the use of digital monitoring tools could facilitate access to these interventions in populations with limited resources. Inconsistent reporting of adverse events represents another methodological limitation. To improve the assessment of the safety profile of RT, it is suggested that uniform definitions, structured event collection systems, and international guidelines for reporting be used. In addition, we recommend prospective registration of protocols on recognized platforms, such as ClinicalTrials.gov or the WHO International Clinical Trials Registry Platform (ICTRP), which would promote transparency, prevent duplication of effort, and strengthen scientific reproducibility. With regard to follow-up, although it is proposed that studies evaluate short-, medium-, and long-term outcomes, it is important to justify this need. BCRL is a chronic condition that can develop months or even years after surgical treatment. Therefore, limited follow-up over time could underestimate the true incidence of the condition and thus overestimate the preventive effect of RT. In addition, it is recommended to consider adopting or developing a Core Outcome Set (COS) specific to BCRL, in order to standardize the outcomes measured and reported in future studies. This strategy will improve comparability across studies, facilitate evidence synthesis, and ensure that patient-relevant outcomes are included. Finally, we emphasize the importance of future studies prioritizing patient-centered outcomes, using robust methodologies and short-, medium-, and long-term follow-up to capture both the early and long-term effects of RT as a preventive strategy for BCRL. Future studies assessing QoL should also report or interpret findings in relation to the minimal clinically important difference (MCID), when validated thresholds are available for the instruments used. Finally, we emphasize the importance of future studies prioritizing patient-centered outcomes, using robust methodologies and short-, medium-, and long-term follow-up to capture both the early and long-term effects of RT as a preventive strategy for BCRL.

### 4.4. Strengths and Limitations

The conclusions of this SR are conditioned by the quantity and quality of the available primary studies. The number of included RCTs was small, and many had small sample sizes, lacked blinding, and presented a high or unclear risk of bias. Heterogeneity in RT protocols, diagnostic criteria for lymphedema, and comparator interventions reduced the clinical and methodological comparability between studies and limited the interpretability of the pooled estimates. As a result, the summary effects may reflect an average across diverse interventions and outcome definitions rather than a single, consistent intervention effect. This heterogeneity also prevented the planned subgroup analyses, as well as most sensitivity analyses, from being performed. In addition, the category of women “at risk” included clinically heterogeneous profiles, particularly in relation to the extent of axillary surgery, radiotherapy exposure, and other treatment-related factors, which limit the applicability of the findings to specific risk subgroups. Another limitation is that the available evidence did not allow a meaningful comparison between RT and other potentially relevant preventive interventions that may promote lymphatic flow, such as aerobic exercise, manual lymphatic drainage, or multimodal physiotherapy approaches. In the included studies, RT was compared mainly with usual care, no structured exercise, or activity restriction, while aerobic exercise appeared as a comparator in only one trial. Therefore, the findings should not be interpreted as establishing the relative clinical value of RT compared with other preventive strategies. In addition, the absence of stratification according to habitual physical activity or baseline activity level should be considered a further limitation. This factor may have acted as an effect modifier, particularly because comparator groups differed across studies and, in some cases, included free physical activity or specific activity targets. As a result, the clinical interpretation of the findings should be made with caution.

A further methodological consideration is that one included RCT enrolled a very small proportion of participants with baseline lymphedema (<2%). Although this corresponded to only three participants and the study was retained to avoid unnecessary loss of relevant information in a field with limited evidence, this decision should be interpreted with caution, as it may slightly attenuate the strictly preventive focus of the review. To explore its potential influence, a sensitivity analysis excluding this trial was performed. The results remained stable after exclusion, suggesting that its inclusion did not materially change the overall direction of the findings. In addition, because fewer than 10 studies were included in each meta-analysis, publication bias could not be reliably assessed, as the methods commonly used to detect it have limited discriminative capacity in this context. Consequently, the possible influence of unpublished studies or small-study effects cannot be ruled out. The CoE was low or very low for most outcomes, mainly due to imprecision and inconsistency in the estimates. Reporting of adverse events was inconsistent, and sociodemographic information was limited, both of which limit the assessment of safety and reduce external validity. Furthermore, although the search was comprehensive and rigorous, the restriction to studies in English, Spanish, or Portuguese may have excluded relevant evidence in other languages.

This SR is the first synthesis to exclusively evaluate RT as a preventive strategy for BCRL in at-risk women. The protocol was registered in PROSPERO and then published in an open-access journal. The methodology was conducted in accordance with the recommendations of the Cochrane Handbook for Systematic Reviews of Interventions and the PRISMA 2020 statement. A comprehensive search was conducted across biomedical databases, the gray literature, trial registries, and preprints, without applying restrictions based on publication date. Study selection, data extraction, and risk of bias assessment were performed independently by peers, with discrepancies resolved by consensus. The risk of bias was assessed using RoB 2.0, and the CoE was assessed using GRADE, presenting Summary of Findings (SoF) tables for clinically relevant outcomes. Patient-centered outcomes were prioritized, including lymphedema (with standardized diagnostic criteria), overall QoL, pain, ROM, grip strength, and adverse events, reinforcing the applicability of the findings in clinical practice.

## 5. Conclusions

The available evidence on RT as a preventive strategy for BCRL remains uncertain. For most of the outcomes evaluated, including the occurrence of lymphedema, arm volume, overall QoL, pain, ROM, and grip strength, the CoE was low or very low, which limits confidence in the findings. Although the preventive effectiveness of RT cannot be established with certainty, the available studies suggest that RT may be a feasible and generally well-tolerated intervention when implemented with professional supervision, gradual load progression, and appropriate clinical monitoring. However, given the low to very low certainty of the evidence and the inconsistent reporting of adverse events, firm conclusions regarding its safety and preventive effectiveness cannot yet be drawn. From a clinical perspective, RT may be viewed as a potentially relevant component within broader preventive or rehabilitation strategies for women at risk of developing BCRL, but any clinical interpretation or recommendation should be made with caution until more robust evidence becomes available. Future RCTs with larger samples, adequate methodological quality, standardized diagnostic criteria, diverse populations, and short-, medium-, and long-term follow-up are needed to more confidently determine the preventive effectiveness of RT and its impact on QoL and functional outcomes.

## 6. Differences Between the Protocol and SR

Although this SR faithfully followed the protocol previously registered in PROSPERO (CRD42023455720) and published in an open-access journal, some minor differences in its execution were identified.

- Follow-up: In the protocol, outcomes were classified as short-, medium-, and long-term. However, in the SR, they were regrouped into two categories: medium term (>3 to 6 weeks) and long term (>6 weeks).
- Definition of adverse events: While the protocol considered only increased lymphedema volume and pain, the RS broadened the definition to include musculoskeletal and general adverse events to more comprehensively assess the safety of the interventions.
- Planned and performed analyses: The protocol considered subgroup analyses by type of RT and by QoL scales. These could not be performed due to the low number of studies per outcome. Instead, sensitivity analyses not previously contemplated in the protocol were performed:
  1. Comparison between different effect measures (SMD vs. MD) for overall QoL.
  2. Application of different diagnostic criteria for lymphedema (≥10%, ≥5–10%, and >3%).
  3. Exclusion of studies with a high risk of bias.
- Inclusion criteria: A specific modification was made to allow an RCT with a low baseline lymphedema rate (<2%). The inclusion was justified because it met the pre-specified requirements of having separate data for eligible participants or representing ≥80% of the total sample.
- Grouping of comparators: The comparators initially proposed were regrouped into three categories: restriction of physical activity, usual care/absence of structured exercise, and structured aerobic exercise.

This improved clinical consistency and comparability of the analyses.

The modifications described do not alter the research question or the study’s objectives. Rather, they are justified as necessary adjustments to align the analysis with the available evidence, ensuring methodological consistency and transparency throughout the process.

## Figures and Tables

**Table 1:** Characteristics of included studies.

Author/Year	Country	Study Design	N (Female)	Stage BC	Treatment	Type of Surgery	Intervention (RT)	Comparator	Sample Size	Age (y) Overall, Mean/Range (SD/SE)	Follow-Up
Ammitzbøll 2019 [43]	Denmark	RCT	158	I; II; III	CT; HT	Lumpectomy; mastectomy	G1: Supervised + self-administered exercise	Control group: Usual care	-G1: 82 -G2: 76	53 ± 10	12 months
Anderson 2012 [44]	USA	RCT	104	I; II; III	CT; RT	Lumpectomy; mastectomy; Sentinel Axillary	RT group: Combined exercise	Control group: Usual care + education	-G1: 52 -G2: 52	53.6 (range: 32–82)	18 months
Courneya 2007 [24]	Canada	RCT	242	I; IIa; IIb; IIIa	CT; HT	Breast conservation	RT group: Supervised resistance training (RET)	G2: Aerobic exercise	-G1: 78 -G2: 82 -G3: 82	49.2 (range 25–78).	6 months
Soriano 2023 [45]	Spain	RCT	60	NR	TH	Tumorectomy/mastectomy; Lymph node resection;	RT group: Supervised training	G3: Usual care	-G1: 32 -G2: 28	52.6 ± 8.8	12 weeks
Kilbreath 2012 [46]	Australia	RCT	160	I; II; III	CT; RT	Mastectomy; axillary node dissection	RT group: Resistance + stretching	Control group: Free physical activity	-G1: 81 -G2: 79	53.5 ± 12.1	6 months
Schmitz 2010 [26]	USA	RCT	154	Ductal carcinoma in situ; I; II; III	CT; RT	Nodes removed	RT group: Progressive weightlifting	Control group: Education + unsupervised exercises	-G1: 77 -G2: 77	56 ± 8	12 months
Schmidt 2017 [47]	Germany	RCT	62	NR	RT	Modified radical mastectomy; subcutaneous mastectomy; breast-conserving surgery	RT group: Arm ergometer (strength + endurance)	Control group: No structured exercise	-G1: 21 -G2: 28	61.6 ± 10	12 weeks
Sagen 2009 [48]	Norway	RCT	207	NR	RT; CT; AHT	Nodes removed; metastasized nodes; breast ablation; breast conserving; surgery, dominant side	RT group: No activity restriction	Control group: Usual care without exercise	-G1: 104 -G2: 100	55 ± 10	24 months

BC: breast cancer; RCT: randomized clinical trial; CT: chemotherapy; HT: hormone therapy; NR: not reported; RT: resistance training; SD: standard deviation; SE: standard error; AHT: anti-hormone treatment; G: group.

**Table 2:** Description of resistance training interventions and comparators in the included studies.

Study	RT Intervention	Duration	Frequency	Intensity/Progression	Supervision	Comparator	Comparator Classification
Sagen 2009 [48]	Progressive RT without restrictions	6 months	2–3 sessions/week	Gradual progression (not specified)	Full	Supervised physical activity restriction	1. Activity restriction
Ammitzbøll 2019 [43]	Combined RT: supervised (phase 1) + home-based (phase 2)	50 weeks	2–3 sessions/week	Initial 7RM, adjusted monthly based on fatigue	Partial	No structured intervention	2. Usual care
Schmitz 2010 [26]	Progressive weightlifting	12 months	2 sessions/week	Gradual progression (not specified)	Partial	Usual care	2. Usual care
Anderson 2012 [44]	Combined RT with walking and education	18 months	2 sessions/week	50–60% 1RM, gradual progression	Partial	Usual care with occasional education	2. Usual care
Kilbreath 2012 [46]	Weekly RT + stretching + home exercises	8 weeks	1 session/week + daily home exercises	Not specified	Partial	Basic education and unsupervised exercises	2. Usual care
Courneya 2007 [24]	Supervised RT	17 weeks	3 sessions/week	G1: 60–70% 1RM G2: 60–80% VO_2_max	Full	G2: Aerobic exercise G3: Usual care	3. Aerobic 2. Usual care
Soriano 2023 [45]	Supervised progressive RT	12 weeks	2 sessions/week	40–70% 1RM, weekly progression	Full	Daily physical activity (≥10,000 steps)	2. Usual care
Schmidt 2017 [47]	Arm-ergometer RT (strength + endurance)	12 weeks	2 sessions/week	60 min/session, intensity according to tolerance	Full	Usual care without supervised training	2. Usual care

RT: resistance training; RM: repetition maximum; VO_2_max: maximal oxygen uptake; G: group.

**Table 3:** Methods and indicators used to assess lymphedema in the included studies.

Author/Year	Evaluation Instrument	Type of Measurement	Assessment Time Points	Operational Definition	Formula Used	Diagnostic Criterion and Outcome Type
Sagen 2009 [48]	SWDI	Inter-limb volume difference (mL)	Baseline, 3, 6, and 24 months	Clinically significant increase in the affected arm	Voldiff = VA − VC; % diff = (VA − VC)/VC) × 100	Dichotomous outcome: ≥10% inter-limb volume increase (water displacement)
Anderson 2012 [44]	Water displacement	Absolute volume (mL)	Every 3 months up to 18 months	Absolute increase relative to baseline value	Mean of two measurements (immersion–withdrawal)	Continuous outcome: no diagnostic cut-off applied
Ammitzbøll 2019 [43]	Water displacement	ILVD (inter-limb volume difference, %)	Postoperative baseline and 12 months	Subclinical lymphedema defined as >3% ILVD	((VA − VC)/VC) × 100	Dichotomous outcome: >3% inter-limb volume increase
Schmitz 2010 [26]	Water displacement	Percentage inter-limb volume difference	Baseline and 12 months	≥5% increase in volume of the affected arm	((VA − VC)/VC) × 100	Dichotomous outcome: ≥5% inter-limb volume increase
Kilbreath 2012 [46]	Tape measure + bioimpedance	Volume estimated using geometric formula	Post-intervention and 6 months	Inter-limb volume difference calculated from circumference measurements	Volume estimated from circumference measurements	Dichotomous outcome: ≥10% inter-limb increase (circumference-based)
Courneya 2007 [24]	Water displacement	Inter-limb volume difference (mL)	Baseline and post-intervention	≥200 mL increase between affected and contralateral arm	Voldiff = VA − VC	Dichotomous outcome: ≥200 mL inter-limb difference

SWDI: single water displacement instrument; ILVD: inter-limb volume difference; VA: volume of the affected arm; VC: volume of the contralateral arm; Voldiff: volume difference; mL: milliliters; % diff: percentage difference.

**Table 4:** Instruments and QoL dimensions assessed in the included studies.

Author/Year	Instrument Used	QoL Dimensions Assessed
Anderson 2012 [44]	FACT-B	Total quality of life
Schmidt 2017 [47]	EORTC QLQ C30	Total quality of life
Soriano 2023 [45]	FACT-B	Total quality of life

FACT-B: Functional Assessment of Cancer Therapy-Breast; EORTC QLQ-C30: European Organisation for Research and Treatment of Cancer Quality of Life Questionnaire Core 30.

**Table 5:** Instruments and indicators used to assess secondary outcomes in the included studies.

Author/Year	Instrument Used	Secondary Outcome Assessed
Sagen 2009 [48]	Visual Analog Scale	Pain intensity
Kilbreath 2012 [46]	Digital inclinometer	ROM: extension, abduction, and external rotation
Ammitzbøll 2019 [43]	Goniometer	ROM: flexion, abduction, and external rotation
Soriano 2023 [45]	Electrogoniometer	Shoulder flexion ROM; handgrip strength
Courneya 2007 [24]	Clinical report	Adverse events
Schmitz 2010 [26]	Session safety log	Adverse events
Ammitzbøll 2019 [43]	Self-report/clinical monitoring	Adverse events
Soriano 2023 [45]	Supervised intervention log	Adverse events
Sagen 2009 [48]	Clinical report	Adverse events
Anderson 2012 [44]	Clinical report/safety record	Adverse events

## Data Availability

The raw data supporting the conclusions of this article will be made available by the authors upon request.

## Supplementary Materials

The following supporting information can be downloaded at https://www.mdpi.com/article/10.3390/jcm15093297/s1. Supplement S1: PRISMA 2020 Main Checklist; Supplement S2: PRISMA 2020 Abstract Checklist; Supplement S3: Search strategy used in each database; Supplement S4: Characteristics of excluded studies; Supplement S5: Characteristics and identification of studies retrieved through other methods; Supplement S6: Risk of bias in the included studies; Supplement S7: Summary of findings (SoF) table for comparison 1: resistance training vs. activity restriction; Supplement S8: Summary of findings (SoF) table for comparison 2: resistance training vs. usual care/no structured exercise; Supplement S9: Summary of findings (SoF) table for comparison 3: resistance training vs. aerobic training; Supplement S10: Risk of bias in sensitivity analysis according to effect measure (SMD vs. MD in overall quality of life); Supplement S11: Certainty of evidence assessment (GRADE) in sensitivity analysis according to effect measure (SMD vs. MD in overall quality of life); Supplement S12: Risk of bias in sensitivity analysis according to diagnostic criteria for lymphedema; Supplement S13: Certainty of evidence assessment (GRADE) in sensitivity analysis according to diagnostic criteria for lymphedema; Supplement S14: Risk of bias in sensitivity analysis excluding studies at high risk of bias; Supplement S15: Certainty of evidence assessment (GRADE) in sensitivity analysis excluding studies at high risk of bias.

## Abbreviations

The following abbreviations are used in this manuscript:

BC	Breast cancer
BCRL	Breast cancer-related lymphedema
QoL	Quality of life
RT	Resistance training
CoE	Certainty of the evidence
ROM	Range of motion
RCT	Randomized clinical trials
SR	Systematic reviews
APTA	American Physical Therapy Association
ACSM	American College of Sports Medicine
VAS	Visual Analog Scale
CI	Confidence intervals
MD	Mean differences
SMD	Standardized mean difference
GRADE	Grading of Recommendations, Assessment, Development and Evaluation
NR	Not reported
SE	Standard error
AHT	Anti-hormone treatment
RM	Repetition maximum
VO_2_max	Maximal oxygen uptake
SWDI	Single water displacement instrument
ILVD	Inter-limb volume difference
VA	Volume of the affected arm
VC	Volume of the contralateral arm
Voldiff	Volume difference
Ml	Milliliters
% diff	Percentage difference
FACT-B	Functional Assessment of Cancer Therapy-Breast
EORTC QLQ-C30	European Organisation for Research and Treatment of Cancer Quality of Life Questionnaire Core 30
