# Supplementary material for: Can Resistance Training Prevent Breast Cancer-Related Lymphedema? A Systematic Review with Meta-Analysis
Source: J Clin Med. 2026 Apr 26;15(9):3297. doi: 10.3390/jcm15093297 (PMC13164433; doi:10.3390/jcm15093297)
Supplement: Supplementary file 1 [file jcm-15-03297-s001.zip › jcm-4230926-supplementary-update.pdf]

Supplemental material

Can resistance training prevent breast cancer-related lymphedema? A systematic review with meta-analysis

Table of contents

Can resistance training prevent breast cancer-related lymphedema? A systematic review with meta-analysis 1
Supplement S1: PRISMA 2020 main checklist 3
Supplement S2: PRISMA 2020 Abstract Checklist 6
Supplement S3: Search strategy used in each database 8
Supplement S4: Characteristics of excluded studies 12
Supplement S5: Characteristics and identification of studies retrieved through other methods 16
Supplement S6: Risk of bias in the included studies 19
Supplement S7: Summary of findings (SoF) table for comparison 1: resistance training vs. activity restriction 27
Supplement S8. Summary of findings (SoF) table for comparison 2: resistance training vs. usual care/no structured exercise 29
Supplement S9: Summary of findings (SoF) table for comparison 3: resistance training vs. aerobic training 36
Supplement S10. Risk of bias in sensitivity analysis according to effect measure (SMD vs. MD in overall quality of life) 37
Supplement S11. Certainty of evidence assessment (GRADE) in sensitivity analysis according to effect measure (SMD vs. MD in overall quality of life) 38
Supplement S12. Risk of bias in sensitivity analysis according to diagnostic criteria for lymphedema 39
Supplement S13. Certainty of evidence assessment (GRADE) in sensitivity analysis according to diagnostic criteria for lymphedema 41
Supplement S14. Risk of bias in sensitivity analysis excluding studies at high risk of bias 44
Supplement S15. Certainty of evidence assessment (GRADE) in sensitivity analysis excluding studies at high risk of bias 46

Supplement S1: PRISMA 2020 main checklist

Section and Topic	Item #	Checklist item	Location where item is reported
**TITLE**			
Title	1	Identify the report as a systematic review.	2-3
**ABSTRACT**			
Abstract	2	See the PRISMA 2020 for Abstracts checklist.	39-58
**INTRODUCTION**			
Rationale	3	Describe the rationale for the review in the context of existing knowledge.	65-100
--Objectives	4	Provide an explicit statement of the objective(s) or question(s) the review addresses.	102-105
**METHODS**			
Eligibility criteria	5	Specify the inclusion and exclusion criteria for the review and how studies were grouped for the syntheses.	114-173
Information sources	6	Specify all databases, registers, websites, organisations, reference lists and other sources searched or consulted to identify studies. Specify the date when each source was last searched or consulted.	179-190
Search strategy	7	Present the full search strategies for all databases, registers and websites, including any filters and limits used.	Suppl S3
Selection process	8	Specify the methods used to decide whether a study met the inclusion criteria of the review, including how many reviewers screened each record and each report retrieved, whether they worked independently, and if applicable, details of automation tools used in the process.	192-198
Data collection process	9	Specify the methods used to collect data from reports, including how many reviewers collected data from each report, whether they worked independently, any processes for obtaining or confirming data from study investigators, and if applicable, details of automation tools used in the process.	199-204
Data items	10a	List and define all outcomes for which data were sought. Specify whether all results that were compatible with each outcome domain in each study were sought (e.g. for all measures, time points, analyses), and if not, the methods used to decide which results to collect.	120-194
	10b	List and define all other variables for which data were sought (e.g. participant and intervention characteristics, funding sources). Describe any assumptions made about any missing or unclear information.	199-204
Study risk of bias assessment	11	Specify the methods used to assess risk of bias in the included studies, including details of the tool(s) used, how many reviewers assessed each study and whether they worked independently, and if applicable, details of automation tools used in the process.	206-215
Effect measures	12	Specify for each outcome the effect measure(s) (e.g. risk ratio, mean difference) used in the synthesis or presentation of results.	225-234
Synthesis methods	13a	Describe the processes used to decide which studies were eligible for each synthesis (e.g. tabulating the study intervention characteristics and comparing against the planned groups for each synthesis (item #5)).	236-243
	13b	Describe any methods required to prepare the data for presentation or synthesis, such as handling of missing summary statistics, or data conversions.	251-268

Section and Topic	Item #	Checklist item	Location where item is reported
	13c	Describe any methods used to tabulate or visually display results of individual studies and syntheses.	251-268
	13d	Describe any methods used to synthesize results and provide a rationale for the choice(s). If meta-analysis was performed, describe the model(s), method(s) to identify the presence and extent of statistical heterogeneity, and software package(s) used.	251-268
	13e	Describe any methods used to explore possible causes of heterogeneity among study results (e.g. subgroup analysis, meta-regression).	270-274
	13f	Describe any sensitivity analyses conducted to assess robustness of the synthesized results.	276-282
Reporting bias assessment	14	Describe any methods used to assess risk of bias due to missing results in a synthesis (arising from reporting biases).	251-282
Certainty assessment	15	Describe any methods used to assess certainty (or confidence) in the body of evidence for an outcome.	284-295
**RESULTS**			
Study selection	16a	Describe the results of the search and selection process, from the number of records identified in the search to the number of studies included in the review, ideally using a flow diagram.	298-306
	16b	Cite studies that might appear to meet the inclusion criteria, but which were excluded, and explain why they were excluded.	Suppl S4-S5
Study characteristics	17	Cite each included study and present its characteristics.	310-331
Risk of bias in studies	18	Present assessments of risk of bias for each included study.	436-451
Results of individual studies	19	For all outcomes, present, for each study: (a) summary statistics for each group (where appropriate) and (b) an effect estimate and its precision (e.g. confidence/credible interval), ideally using structured tables or plots.	453-551
Results of syntheses	20a	For each synthesis, briefly summarise the characteristics and risk of bias among contributing studies.	453-551
	20b	Present results of all statistical syntheses conducted. If meta-analysis was done, present for each the summary estimate and its precision (e.g. confidence/credible interval) and measures of statistical heterogeneity. If comparing groups, describe the direction of the effect.	453-551
	20c	Present results of all investigations of possible causes of heterogeneity among study results.	453-551
	20d	Present results of all sensitivity analyses conducted to assess the robustness of the synthesized results.	453-551
Reporting biases	21	Present assessments of risk of bias due to missing results (arising from reporting biases) for each synthesis assessed.	453-551
Certainty of evidence	22	Present assessments of certainty (or confidence) in the body of evidence for each outcome assessed.	453-551
**DISCUSSION**			
Discussion	23a	Provide a general interpretation of the results in the context of other evidence.	552-573

Section and Topic	Item #	Checklist item	Location where item is reported
	23b	Discuss any limitations of the evidence included in the review.	553-573
	23c	Discuss any limitations of the review processes used.	575-690
	23d	Discuss implications of the results for practice, policy, and future research.	575-690
**OTHER INFORMATION**			
Registration and protocol	24a	Provide registration information for the review, including register name and registration number, or state that the review was not registered.	57-58
	24b	Indicate where the review protocol can be accessed, or state that a protocol was not prepared.	107-112
	24c	Describe and explain any amendments to information provided at registration or in the protocol.	692-721
Support	25	Describe sources of financial or non-financial support for the review, and the role of the funders or sponsors in the review.	748
Competing interests	26	Declare any competing interests of review authors.	764
Availability of data, code and other materials	27	Report which of the following are publicly available and where they can be found: template data collection forms; data extracted from included studies; data used for all analyses; analytic code; any other materials used in the review.	757

From: Page MJ, McKenzie JE, Bossuyt PM, Boutron I, Hoffmann TC, Mulrow CD, et al. The PRISMA 2020 statement: an updated guideline for reporting systematic reviews. BMJ 2021;372:n71. doi: 10.1136/bmj.n71. This work is licensed under CC BY 4.0. To view a copy of this license, visit https://creativecommons.org/licenses/by/4.0/

Supplement S2: PRISMA 2020 Abstract Checklist

Section and Topic	Item #	Checklist item	Reported (Yes/No)
**TITLE**			
Title	1	Identify the report as a systematic review.	Yes
**BACKGROUND**			
Objectives	2	Provide an explicit statement of the main objective(s) or question(s) the review addresses.	Yes
**METHODS**			
Eligibility criteria	3	Specify the inclusion and exclusion criteria for the review.	Yes
Information sources	4	Specify the information sources (e.g. databases, registers) used to identify studies and the date when each was last searched.	Yes
Risk of bias	5	Specify the methods used to assess risk of bias in the included studies.	Yes
Synthesis of results	6	Specify the methods used to present and synthesise results.	Yes
**RESULTS**			
Included studies	7	Give the total number of included studies and participants and summarise relevant characteristics of studies.	Yes
Synthesis of results	8	Present results for main outcomes, preferably indicating the number of included studies and participants for each. If meta-analysis was done, report the summary estimate and confidence/credible interval. If comparing groups, indicate the direction of the effect (i.e. which group is favoured).	Yes
**DISCUSSION**			

Section and Topic	Item #	Checklist item	Reported (Yes/No)
Limitations of evidence	9	Provide a brief summary of the limitations of the evidence included in the review (e.g. study risk of bias, inconsistency and imprecision).	Yes
Interpretation	10	Provide a general interpretation of the results and important implications.	Yes
**OTHER**			
Funding	11	Specify the primary source of funding for the review.	Yes
Registration	12	Provide the register name and registration number.	Yes

From: Page MJ, McKenzie JE, Bossuyt PM, Boutron I, Hoffmann TC, Mulrow CD, et al. The PRISMA 2020 statement: an updated guideline for reporting systematic reviews. BMJ 2021;372:n71. doi: 10.1136/bmj.n71. This work is licensed under CC BY 4.0. To view a copy of this license, visit https://creativecommons.org/licenses/by/4.0/

Supplement S3: Search strategy used in each database

Strategy for the Medline/PubMed database

Number	Search terms
#1	"Breast Neoplasms"[Mesh]
#2	"Breast Neoplasms"
#3	breast cancer*[tiab]
#4	breast tumor*[tiab]
#5	breast tumour*[tiab]
#6	mammary neoplasm*[tiab]
#7	mammary carcinoma*[tiab]
#8	breast neoplasm*[tiab]
#9	breast carcinoma*[tiab]
#10	or/#1-9
#11	Breast Cancer Lymphedema"[Mesh]
#12	"Breast Cancer Lymphedema"
#13	Breast Cancer Treatment-Related Lymphedema
#14	or/#11-13
#15	prevention and control
#16	"prevention and control" [Subheading]
#17	"Preventive Health Services"[Mesh]
#18	"Early Diagnosis"[Mesh]
#19	"Risk"[Mesh]
#20	or/#15-19
#21	"Resistance Training"[Mesh]
#22	"Resistance Training"
#23	Exercise Program, Weight-Lifting
#24	Weight Lifting Exercise Program
#25	strength exercise
#26	weight training
#27	weight lifting
#28	resistance exercise
#29	physical therapy techniques
#30	Physical Therapy Specialty
#31	REHABILITATION
#32	Physiotherapy
#33	or/#21-32
#34	clinical[Title/Abstract]
#35	trial[Title/Abstract]
#36	clinical trials as topic[MeSH Terms]
#37	clinical trial[Publication Type]
#38	random*[Title/Abstract]
#39	random allocation[MeSH Terms]
#40	therapeutic use[MeSH Subheading]
#41	or/#34-40
#42	and/#10, 14, 20, 33

Search strategy used on Lilacs:

Number	Search terms
#1	neoplasias mamarias
#2	cancer de seno
#3	or/#1-2
#4	linfedema del cáncer de mama
#5	linfedema posmastectomía
#6	linfedema relacionado con el tratamiento del cáncer de mama
#7	or/#4-6
#8	servicios preventivos de salud
#9	prevención y promoción de la salud
#10	salud preventive
#11	or/#8-10
#12	entrenamiento de fuerza
#13	Musculación
#14	programa de fortalecimiento levantando peso
#15	levantamiento de peso
#16	modalidades de fisioterapia
#17	fisioterapia
#18	especialidad de fisioterapia
#19	especialidad de terapia física
#20	Rehabilitación
#21	or/#12-20
#22	type_of_study:("clinical_trials")
#23	instance:"regional"
#24	and/#3, 7, 11, 21
#25	and/#21, 22, 23

Search strategy used on PEDro:

Abstract & Title: Risk breast cancer-related lymphedema
Therapy: strength training
Problem: oedema
Body Part: -
Subdiscipline: oncology
Topic: -
Method: clinical trial
Autor/Association: -
Title Only: -
Source: -
Published Since: -
New records added since: -
Score at least: -
When Searching: Match all search term (AND)

Search strategy used on Cochrane Central Register of Controlled Trials:

Number	Seach terms
#1	MeSH descriptor: [Breast Neoplasms] explode all trees
#2	Breast Neoplasms
#3	breast cancer*
#4	mammary neoplasm*
#5	MeSH descriptor: [Breast Cancer Lymphedema] explode all trees
#6	breast cancer-related lymphedema
#7	Breast Cancer Treatment-Related Lymphedema
#8	prevention and control
#9	MeSH descriptor: [Preventive Health Services] explode all trees
#10	MeSH descriptor: [Early Diagnosis] explode all trees
#11	MeSH descriptor: [Risk] explode all trees
#12	#1 OR #2 OR #3 OR #4
#13	#5 OR #6 OR #7
#14	#8 OR #9 OR #10 OR #11
#15	MeSH descriptor: [Resistance Training] explode all trees
#16	Resistance Training
#17	Exercise Program, Weight-Lifting
#18	Weight Lifting Exercise Program
#19	strength exercise
#20	resistance exercise
#21	physical therapy techniques
#22	Physical Therapy Specialty
#23	REHABILITATION
#24	MeSH descriptor: [Physical Therapy Specialty] explode all trees
#25	#12 AND #13 AND #14
#26	#15 OR #16 OR #17 OR #18 OR #19 OR #20 OR #21 OR #22 OR #23 OR #24
#27	#25 AND #26

Search strategy used on Embase:

Number	Search terms
#1	'breast tumor'/exp
#2	breast AND neoplasms
#3	'breast cancer'
#4	#1 OR #2 OR #3
#5	'breast cancer-related lymphedema'/exp
#6	'breast cancer-related lymphedema'/syn
#7	#5 OR #6
#8	('prevention'/exp OR prevention) AND ('control'/exp OR control)
#9	'preventive health service'/exp
#10	'early diagnosis'/exp

#11	'risk'/exp
#12	#8 OR #9 OR #10 OR #11
#13	'resistance training'/exp
#14	'weight lifting'/exp
#15	'strength exercise'/exp
#16	'weight training'/exp
#17	'physiotherapy'/exp
#18	'rehabilitation'/exp
#19	#13 OR #14 OR #15 OR #16 OR #17 OR #18
#20	#7 AND #12 AND #19
#21	'randomized controlled trial'/exp
#22	#20 AND #21

Supplement S4: Characteristics of excluded studies

Study [ref]	Design	Country	N	Reasons for exclusion
Lund 2019 [1]	Randomized clinical trial	Denmark	82	Wrong outcome
Szuba 2016 [2]	Randomized clinical trial	Poland	44	Wrong intervention
Vignes 2013 [3]	Other research design	France	129	Wrong population/Wrong design
Harvie 2024 [4]	Randomized clinical trial	UK	57	Wrong population
Winkels 2017 [5]	Randomized clinical trial	USA	351	Wrong population
De Vrieze 2022 [6]	Randomized clinical trial	Belgium	194	Wrong population
Koelmeyer 2022 [7]	Randomized clinical trial	USA	918	Wrong intervention
Muñoz-Alcaraz 2020 [8] (8)	Randomized clinical trial - Protocol	Spain	---	Wrong design - Protocol
Dönmez 2017 [9]	Randomized clinical trial	Turkey	52	Wrong intervention
Zhang 2017 [10]	Randomized clinical trial	USA	141	Wrong population
Ridner 2019 [11]	Randomized clinical trial – interim analysis	USA	508	Wrong intervention
Cho 2016 [12]	Randomized clinical trial	South Korea	48	Wrong intervention
Temur 2019 [13]	Randomized clinical trial	Turkey	61	Wrong intervention
De Oliveira 2018 [14]	Clinical trial	Poland	106	Wrong design
Torres-Lacomba 2010 [15]	Randomized clinical trial	Spain	120	Wrong intervention
Stubblefield 2017 [16]	Literature review	USA	---	Wrong study design
Byun 2021 [17]	Retrospective analysis	Korea	5549	Wrong study design
Wenczl 2016 [18]	Case report	Hungary	---	Wrong population/language
Bakar 2018 [19]	Other research design	Turkey	63	Wrong intervention
De Oliveira 2014 [20]	Non-randomized controlled clinical trial	Brazil	89	Wrong study design
Hayes 2009 [21]	Randomized clinical trial	Australia	32	Wrong population
Kilgore 2018 [22]	Other research design	USA	146	Wrong intervention/Wrong study design
Smoot 2016 [23]	Prospective longitudinal study	USA	380	Wrong study design
Arinaga 2019 [24]	Randomized clinical trial	Japan	43	Wrong population
Grabenbauer 2016 [25]	Clinical trial	Germany	45	Wrong study design
Sánchez 2015 [26]	Randomized clinical trial	Spain	153	Wrong intervention
Fu 2014 [27]	Quasi-experimental	USA	140	Wrong study design
Box 2002 [28]	Randomized clinical trial	Australia	65	Wrong intervention

Rizzi 2020 [29]	Randomized clinical trial	Brazil	60	Wrong intervention
Szolnoky 2009 [30]	Randomized clinical trial	Hungary	17	Wrong intervention
Devoogdt 2011 [31]	Randomized clinical trial	Belgium	160	Wrong intervention
Rezende 2006 [32]	Randomized clinical trial	Brazil	60	Wrong intervention
Castro-Sánchez 2011 [33]	Randomized clinical trial	Spain	48	Wrong intervention
Ammitzbøll 2019 [34]	Randomized clinical trial	Denmark	45	Wrong study design
Bloomquist 2019 [35]	Randomized clinical trial	Denmark	153	Wrong intervention
Ahmed 2006 [36]	Randomized clinical trial	USA	85	Wrong Outcome

References:

1. Lund, L.W.; Ammitzbøll, G.; Hansen, D.G.; Andersen, E.A.W.; Dalton, S.O. Adherence to a long-term progressive resistance training program, combining supervised and home-based exercise for breast cancer patients during adjuvant treatment. Acta Oncol. 2019, 58, 650–657.
2. Szuba, A.; Chacaj, A.; Koba-Wszedybyl, M.; Hawro, R.; Jasinski, R.; Tarkowski, R.; Bebenek, M.; Szewczyk, K.; Forgacz, J.; Jodkowska, A.; et al. Upper extremity lymphedema after axillary lymph node dissection: Prospective lymphoscintigraphic evaluation. Lymphology 2016, 49, 44–56.
3. Vignes, S.; Blanchard, M.; Arrault, M.; Porcher, R. Intensive complete decongestive physiotherapy for cancer-related upper-limb lymphedema: 11 days achieved greater volume reduction than 4. Gynecol. Oncol. 2013, 131, 127–130.
4. Harvie, M.; Livingstone, K.; McMullan, D.; Pegington, M.; Lombardelli, C.; Adams, J.; Farragher, M.; Barrett, E.; Bundred, N. BE-WEL trial (breast: evaluation of weight and exercise for lymphoedema) testing weight control and exercise programmes for women with breast cancer related lymphoedema: A feasibility trial. Breast Cancer Res. Treat. 2024, 207, 203–212.
5. Winkels, R.M.; Sturgeon, K.M.; Kallan, M.J.; Dean, L.T.; Zhang, Z.; Evangelisti, M.; Brown, J.C.; Sarwer, D.B.; Troxel, A.B.; Denlinger, C.; et al. The Women In Steady Exercise Research (WISER) survivor trial: The innovative transdisciplinary design of a randomized controlled trial of exercise and weight-loss interventions among breast cancer survivors with lymphedema. Contemp. Clin. Trials 2017, 61, 63–72.
6. De Vrieze, T.; Gebruers, N.; Nevelsteen, I.; Thomis, S.; De Groef, A.; Tjalma, W.A.A.; Belgrado, J.P.; Vandermeeren, L.; Monten, C.; Hanssens, M.; et al. Does manual lymphatic drainage add value in reducing suprafascial fluid accumulation and skin elasticity in patients with breast cancer-related lymphedema? Phys. Ther. 2022, 102, pzac137.
7. Koelmeyer, L.A.; Gaitatzis, K.; Dietrich, M.S.; Shah, C.S.; Boyages, J.; McLaughlin, S.A.; Taback, B.; Stolldorf, D.P.; Elder, E.; Hughes, T.M.; et al. Risk factors for breast cancer-related lymphedema in patients undergoing 3 years of prospective surveillance with intervention. Cancer 2022, 128, 3408–3415.
8. Muñoz-Alcaraz, M.N.; Pérula-de-Torres, L.Á.; Serrano-Merino, J.; Jiménez-Vílchez, A.J.; Olmo-Carmona, M.V.; Muñoz-García, M.T.; Bartolomé-Moreno, C.; Oliván-Blázquez, B.; Magallón-Botaya, R. Efficacy and efficiency of a new therapeutic approach based on activity-oriented proprioceptive antiedema therapy (TAPA) for edema reduction and improved occupational performance in the rehabilitation of breast cancer-related arm lymphedema in women: A controlled, randomized clinical trial. BMC Cancer 2020, 20, 1074.
9. Dönmez, A.A.; Kapucu, S. The effectiveness of a clinical and home-based physical activity program and simple lymphatic drainage in the prevention of breast cancer-related lymphedema: A prospective randomized controlled study. Eur. J. Oncol. Nurs. 2017, 31, 12–21.
10. Zhang, X.; Brown, J.C.; Paskett, E.D.; Zemel, B.S.; Cheville, A.L.; Schmitz, K.H. Changes in arm tissue composition with slowly progressive weight-lifting among women with breast cancer-related lymphedema. Breast Cancer Res. Treat. 2017, 164, 79–88.
11. Ridner, S.H.; Dietrich, M.S.; Cowher, M.S.; Taback, B.; McLaughlin, S.A.; Ajkay, N.; Boyages, J.; Koelmeyer, L.; DeSnyder, S.; Wagner, J.; et al. A randomized trial evaluating bioimpedance spectroscopy versus tape measurement for the prevention of lymphedema following treatment for breast cancer: Interim analysis. Ann. Surg. Oncol. 2019, 26, 3250–3259.

12. Cho, Y.; Do, J.; Jung, S.; Kwon, O.; Jeon, J.Y. Effects of a physical therapy program combined with manual lymphatic drainage on shoulder function, quality of life, lymphedema incidence, and pain in breast cancer patients with axillary web syndrome following axillary dissection. Support. Care Cancer 2016, 24, 2047–2057.
13. Temur, K.; Kapucu, S. The effectiveness of lymphedema self-management in the prevention of breast cancer-related lymphedema and quality of life: A randomized controlled trial. Eur. J. Oncol. Nurs. 2019, 40, 22–35.
14. De Oliveira, M.M.F.; Gurgel, M.S.C.; Amorim, B.J.; Ramos, C.D.; Derchain, S.; Furlan-Santos, N.; Dos Santos, C.C.; Sarian, L.O. Long term effects of manual lymphatic drainage and active exercises on physical morbidities, lymphoscintigraphy parameters and lymphedema formation in patients operated due to breast cancer: A clinical trial. PLoS ONE 2018, 13, e0189176.
15. Torres Lacomba, M.; Yuste Sánchez, M.J.; Zapico Goñi, A.; Prieto Merino, D.; Mayoral del Moral, O.; Cerezo Téllez, E.; Minayo Mogollón, E. Effectiveness of early physiotherapy to prevent lymphoedema after surgery for breast cancer: Randomised, single blinded, clinical trial. BMJ 2010, 340, b5396.
16. Stubblefield, M.D. The underutilization of rehabilitation to treat physical impairments in breast cancer survivors. PM R 2017, 9, S317–S323.
17. Byun, H.K.; Chang, J.S.; Im, S.H.; Kirova, Y.M.; Arsene-Henry, A.; Choi, S.H.; Cho, Y.U.; Park, H.S.; Kim, J.Y.; Suh, C.O.; et al. Risk of lymphedema following contemporary treatment for breast cancer: An analysis of 7617 consecutive patients from a multidisciplinary perspective. Ann. Surg. 2021, 274, 170–178.
18. Wenczl, E. Daganatos betegekben kialakult másodlagos nyiroködéma ellátása [Management of secondary lymphedema in patients with cancer]. Orv. Hetil. 2016, 157, 488–494.
19. Bakar, Y.; Tuğral, A.; Üyetürk, Ü. Measurement of local tissue water in patients with breast cancer-related lymphedema. Lymphat. Res. Biol. 2018, 16, 160–164.
20. de Oliveira, M.M.; de Rezende, L.F.; do Amaral, M.T.; Pinto e Silva, M.P.; Morais, S.S.; Gurgel, M.S. Manual lymphatic drainage versus exercise in the early postoperative period for breast cancer. Physiother. Theory Pract. 2014, 30, 384–389.
21. Hayes, S.C.; Reul-Hirche, H.; Turner, J. Exercise and secondary lymphedema: Safety, potential benefits, and research issues. Med. Sci. Sports Exerc. 2009, 41, 483–489.
22. Kilgore, L.J.; Korentager, S.S.; Hangge, A.N.; Amin, A.L.; Balanoff, C.R.; Larson, K.E.; Mitchell, M.P.; Chen, J.G.; Burgen, E.; Khan, Q.J.; et al. Reducing breast cancer-related lymphedema (BCRL) through prospective surveillance monitoring using bioimpedance spectroscopy (BIS) and patient directed self-interventions. Ann. Surg. Oncol. 2018, 25, 2948–2952.
23. Smoot, B.; Cooper, B.A.; Conley, Y.; Kober, K.; Levine, J.D.; Mastick, J.; Topp, K.; Miaskowski, C. Differences in limb volume trajectories after breast cancer treatment. J. Cancer Surviv. 2016, 10, 772–782.
24. Arinaga, Y.; Piller, N.; Sato, F.; Ishida, T.; Ohtake, T.; Kikuchi, K.; Sato-Tadano, A.; Tada, H.; Miyashita, M. The 10-min holistic self-care for patients with breast cancer-related lymphedema: Pilot randomized controlled study. Tohoku J. Exp. Med. 2019, 247, 139–147.
25. Grabenbauer, A.; Grabenbauer, A.J.; Lengenfelder, R.; Grabenbauer, G.G.; Distel, L.V. Feasibility of a 12-month-exercise intervention during and after radiation and chemotherapy in cancer patients: Impact on quality of life, peak oxygen consumption, and body composition. Radiat. Oncol. 2016, 11, 42.
26. Yuste Sánchez, M.J.; Lacomba, M.T.; Sánchez, B.S.; Merino, D.P.; da Costa, S.P.; Téllez, E.C.; Zapico Goñi, Á. Health related quality of life improvement in breast cancer patients: Secondary outcome from a simple blinded, randomised clinical trial. Breast 2015, 24, 75–81.
27. Fu, M.R.; Axelrod, D.; Guth, A.A.; Cartwright, F.; Qiu, Z.; Goldberg, J.D.; Kim, J.; Scagliola, J.; Kleinman, R.; Haber, J. Proactive approach to lymphedema risk reduction: A prospective study. Ann. Surg. Oncol. 2014, 21, 3481–3489.
28. Box, R.C.; Reul-Hirche, H.M.; Bullock-Saxton, J.E.; Furnival, C.M. Physiotherapy after breast cancer surgery: Results of a randomised controlled study to minimise lymphoedema. Breast Cancer Res. Treat. 2002, 75, 51–64.
29. Rizzi, S.K.L.A.; Haddad, C.A.S.; Elias, S.; Nazário, A.C.P.; Facina, G. Exercise protocol for shoulder movement with restricted amplitude movement for 15 or 30 days after breast-conserving surgery with oncoplastic technique: Randomized clinical trial. Mastology 2020, 30 (Suppl. 1).
30. Szolnoky, G.; Lakatos, B.; Keskeny, T.; Varga, E.; Varga, M.; Dobozy, A.; Kemény, L. Intermittent pneumatic compression acts synergistically with manual lymphatic drainage in complex decongestive physiotherapy for breast cancer treatment-related lymphedema. Lymphology 2009, 42, 188–194.

31. Devoogdt, N.; Christiaens, M.R.; Geraerts, I.; Truijen, S.; Smeets, A.; Leunen, K.; Neven, P.; Van Kampen, M. Effect of manual lymph drainage in addition to guidelines and exercise therapy on arm lymphoedema related to breast cancer: Randomised controlled trial. BMJ 2011, 343, d5326.
32. Rezende, L.F.; Beletti, P.O.; Franco, R.L.; Moraes, S.S.; Gurgel, M.S. Exercícios livres versus direcionados nas complicações pós-operatórias de câncer de mama [Random clinical comparative trial between free and directed exercise in post-operative complications of breast cancer]. Rev. Assoc. Med. Bras. 2006, 52, 37–42.
33. Castro-Sánchez, A.M.; Moreno-Lorenzo, C.; Matarán-Peñarrocha, G.A.; Aguilar-Ferrándiz, M.E.; Almagro-Céspedes, I.; Anaya-Ojeda, J. Preventing lymphoedema after breast cancer surgery by elastic restraint orthotic and manual lymphatic drainage: A randomized clinical trial]. Med. Clin. (Barc.) 2011, 137, 204–207.
34. Ammitzbøll, G.; Dalton, S.O. Mounting evidence supports the safety of weight lifting after breast cancer. Acta Oncol. 2019, 58, 1665–1666.
35. Bloomquist, K.; Adamsen, L.; Hayes, S.C.; Lillelund, C.; Andersen, C.; Christensen, K.B.; Oturai, P.; Ejlertsen, B.; Tuxen, M.K.; Møller, T. Heavy-load resistance exercise during chemotherapy in physically inactive breast cancer survivors at risk for lymphedema: A randomized trial. Acta Oncol. 2019, 58, 1667–1675.
36. Ahmed, R.L.; Thomas, W.; Yee, D.; Schmitz, K.H. Randomized controlled trial of weight training and lymphedema in breast cancer survivors. J. Clin. Oncol. 2006, 24, 2765–2772.

Supplement S5: Characteristics and identification of studies retrieved through other methods

**Study** [ref]	**Design**	**Country**	**N**	**Reasons for exclusion**
Bok 2016 [37]	Randomized clinical trial	Korea	32	Wrong population
Buchan 2016 [38]	Randomized clinical trial	Australia	41	Wrong population
Do 2015 [39]	Randomized clinical trial	South Korea	41	Wrong intervention
Cormie 2013 [40]	Randomized clinical trial	Australia	62	Wrong population
Cormie 2013 [41]	Randomized, crossover design	Australia	17	Wrong population
Cormie 2016 [42]	Randomized, crossover design	Australia	21	Wrong population
Singh 2015 [43]	Randomized, crossover design	Australia	25	Wrong population
Fernández-Lao 2013 [44]	Controlled clinical trial	Spain	98	Wrong study design
Jeon 2017 [45]	Video article	Korea	---	Wrong study design
Johansson 2013 [46]	Randomized clinical trial	Sweden	29	Wrong population
Kim 2010 [47]	Randomized clinical trial	Republic of Korea	40	Wrong population
Letellier 2014 [48]	Randomized clinical trial	Canada	25	Wrong population
Park 2017 [49]	Randomized clinical trial	Republic of Korea	69	Wrong population
Schmitz 2009 [50]	Randomized clinical trial	USA	141	Wrong population
Simonavice 2015 [51]	Pre- and post-intervention	USA	27	Wrong study design
Simonavice 2014 [52]	Pre- and post-intervention	USA	23	Wrong study design
Simonavice 2017 [53]	Pre- and post-intervention	USA	27	Wrong study design
Singh 2016 [54]	Randomized clinical trial	Australia	41	Wrong population
Zhang 2016 [55]	Randomized clinical trial	Republic of China	1000	Wrong intervention

Zhang 2017 [56]	Randomized clinical trial	USA	141	Wrong population
Zimmermann 2012 [57]	Randomized clinical trial	Germany	33	Wrong intervention
Brown 2015 [58]	Randomized clinical trial	USA	295	Wrong population
Brown 2015 [59]	Randomized clinical trial	USA	295	Wrong population
Rezende 2006 [60]	Randomized clinical trial	Brazil	60	Duplicate
Hayes 2013 [61]	Randomized clinical trial	Australia	194	Wrong intervention
Anderson 2012 [62]	Randomized clinical trial	USA	104	Duplicate
Ahmed 2006 [63]	Randomized clinical trial	USA	85	Duplicate
Courneya 2007 [64]	Randomized clinical trial	Canada	242	Duplicate
Kilbreath 2012 [65]	Randomized clinical trial	Australia	160	Duplicate
Schmidt 2017 [66]	Randomized clinical trial	Germany	49	Duplicate
Schmitz 2010 [67]	Randomized clinical trial	USA	134	Duplicate

References:

37. Bok, S.K.; Jeon, Y.; Hwang, P.S. Ultrasonographic evaluation of the effects of progressive resistive exercise in breast can-cer-related lymphedema. Lymphat. Res. Biol. 2016, 14, 18–24.
38. Buchan, J.; Janda, M.; Box, R.; Schmitz, K.; Hayes, S. A randomized trial on the effect of exercise mode on breast can-cer-related lymphedema. Med. Sci. Sports Exerc. 2016, 48, 1866–1874.
39. Do, J.H.; Kim, W.; Cho, Y.K.; Lee, J.; Song, E.J.; Chun, Y.M.; Jeon, J.Y. Effects of resistance exercises and complex deconges-tive therapy on arm function and muscular strength in breast cancer related lymphedema. Lymphology 2015, 48, 184–196.
40. Cormie, P.; Pumpa, K.; Galvão, D.A.; Turner, E.; Spry, N.; Saunders, C.; Zissiadis, Y.; Newton, R.U. Is it safe and efficacious for women with lymphedema secondary to breast cancer to lift heavy weights during exercise: A randomised controlled trial. J. Cancer Surviv. 2013, 7, 413–424
41. Cormie, P.; Galvão, D.A.; Spry, N.; Newton, R.U. Neither heavy nor light load resistance exercise acutely exacerbates lymphedema in breast cancer survivor. Integr. Cancer Ther. 2013, 12, 423–432.
42. Cormie, P.; Singh, B.; Hayes, S.; Peake, J.M.; Galvão, D.A.; Taaffe, D.R.; Spry, N.; Nosaka, K.; Cornish, B.; Schmitz, K.H.; et al. Acute inflammatory response to low-, moderate-, and high-load resistance exercise in women with breast cancer-related lymphedema. Integr. Cancer Ther. 2016, 15, 308–317.
43. Singh, B.; Newton, R.U.; Cormie, P.; Galvao, D.A.; Cornish, B.; Reul-Hirche, H.; Smith, C.; Nosaka, K.; Hayes, S.C. Effects of compression on lymphedema during resistance exercise in women with breast cancer-related lymphedema: A random-ized, cross-over trial. Lymphology 2015, 48, 80–92.
44. Fernández-Lao, C.; Cantarero-Villanueva, I.; Ariza-Garcia, A.; Courtney, C.; Fernández-de-las-Peñas, C.; Arroyo-Morales, M. Water versus land-based multimodal exercise program effects on body composition in breast cancer survivors: A con-trolled clinical trial. Support. Care Cancer 2013, 21, 521–530.
45. Jeon, Y.; Beom, J.; Ahn, S.; Bok, S.K. Ultrasonographic evaluation of breast cancer-related lymphedema. J. Vis. Exp. 2017, 119, 54996.
46. Johansson, K.; Hayes, S.; Speck, R.M.; Schmitz, K.H. Water-based exercise for patients with chronic arm lymphedema: A randomized controlled pilot trial. Am. J. Phys. Med. Rehabil. 2013, 92, 312–319.
47. Kim, D.S.; Sim, Y.J.; Jeong, H.J.; Kim, G.C. Effect of active resistive exercise on breast cancer-related lymphedema: A ran-domized controlled trial. Arch. Phys. Med. Rehabil. 2010, 91, 1844–1848.
48. Letellier, M.E.; Towers, A.; Shimony, A.; Tidhar, D. Breast cancer-related lymphedema: A randomized controlled pilot and feasibility study. Am. J. Phys. Med. Rehabil. 2014, 93, 751–761.

49. Park, J.H. The effects of complex exercise on shoulder range of motion and pain for women with breast cancer-related lymphedema: A single-blind, randomized controlled trial. Breast Cancer 2017, 24, 608–614.
50. Schmitz, K.H.; Ahmed, R.L.; Troxel, A.; Cheville, A.; Smith, R.; Lewis-Grant, L.; Bryan, C.J.; Williams-Smith, C.T.; Greene, Q.P. Weight lifting in women with breast-cancer-related lymphedema. N. Engl. J. Med. 2009, 361, 664–673.
51. Simonavice, E.; Liu, P.Y.; Ilich, J.Z.; Kim, J.S.; Arjmandi, B.H.; Panton, L.B. The effects of resistance training on physical function and quality of life in breast cancer survivors. Healthcare 2015, 3, 695–709.
52. Simonavice, E.; Liu, P.Y.; Ilich, J.Z.; Kim, J.S.; Arjmandi, B.; Panton, L.B. The effects of a 6-month resistance training and dried plum consumption intervention on strength, body composition, blood markers of bone turnover, and inflammation in breast cancer survivors. Appl. Physiol. Nutr. Metab. 2014, 39, 730–739.
53. Simonavice, E.; Kim, J.S.; Panton, L. Effects of resistance exercise in women with or at risk for breast cancer-related lymphedema. Support. Care Cancer 2017, 25, 9–15.
54. Singh, B.; Buchan, J.; Box, R.; Janda, M.; Peake, J.; Purcell, A.; Reul-Hirche, H.; Hayes, S.C. Compression use during an ex-ercise intervention and associated changes in breast cancer-related lymphedema. Asia Pac. J. Clin. Oncol. 2016, 12, 216–224.
55. Zhang, L.; Fan, A.; Yan, J.; He, Y.; Zhang, H.; Zhang, H.; Zhong, Q.; Liu, F.; Luo, Q.; Zhang, L.; et al. Combining manual lymph drainage with physical exercise after modified radical mastectomy effectively prevents upper limb lymphedema. Lymphat. Res. Biol. 2016, 14, 104–108.
56. Zhang, X.; Brown, J.C.; Paskett, E.D.; Zemel, B.S.; Cheville, A.L.; Schmitz, K.H. Changes in arm tissue composition with slowly progressive weight-lifting among women with breast cancer-related lymphedema. Breast Cancer Res. Treat. 2017, 164, 79–88.
57. Zimmermann, A.; Wozniewski, M.; Szklarska, A.; Lipowicz, A.; Szuba, A. Efficacy of manual lymphatic drainage in pre-venting secondary lymphedema after breast cancer surgery. Lymphology 2012, 45, 103–112.
58. Brown, J.C.; Schmitz, K.H. Weight lifting and physical function among survivors of breast cancer: A post hoc analysis of a randomized controlled trial. J. Clin. Oncol. 2015, 33, 2184–2189.
59. Brown, J.C.; Schmitz, K.H. Weight lifting and appendicular skeletal muscle mass among breast cancer survivors: A ran-domized controlled trial. Breast Cancer Res. Treat. 2015, 151, 385–392.
60. de Rezende, L.F.; Franco, R.L.; de Rezende, M.F.; Beletti, P.O.; Morais, S.S.; Gurgel, M.S. Two exercise schemes in postoper-ative breast cancer: Comparison of effects on shoulder movement and lymphatic disturbance. Tumori 2006, 92, 55–61.
61. Hayes, S.C.; Rye, S.; Disipio, T.; Yates, P.; Bashford, J.; Pyke, C.; Saunders, C.; Battistutta, D.; Eakin, E. Exercise for health: A randomized, controlled trial evaluating the impact of a pragmatic, translational exercise intervention on the quality of life, function and treatment-related side effects following breast cancer. Breast Cancer Res. Treat. 2013, 137, 175–186.
62. Anderson, R.T.; Kimmick, G.G.; McCoy, T.P.; Hopkins, J.; Levine, E.; Miller, G.; Ribisl, P.; Mihalko, S.L. A Randomized Trial of Exercise on Well-Being and Function Following Breast Cancer Surgery: The RESTORE Trial. J. Cancer Surviv. 2012, 6, 172–181.
63. Ahmed, R.L.; Thomas, W.; Yee, D.; Schmitz, K.H. Randomized Controlled Trial of Weight Training and Lymphedema in Breast Cancer Survivors. J. Clin. Oncol. 2006, 24, 2765–2772.
64. Courneya, K.S.; Segal, R.J.; Mackey, J.R.; Gelmon, K.; Reid, R.D.; Friedenreich, C.M.; Ladha, A.B.; Proulx, C.; Vallance, J.K.; Lane, K.; et al. Effects of Aerobic and Resistance Exercise in Breast Cancer Patients Receiving Adjuvant Chemotherapy: A Multicenter Randomized Controlled Trial. J. Clin. Oncol. 2007, 25, 4396–4404.
65. Kilbreath, S.L.; Refshauge, K.M.; Beith, J.M.; Ward, L.C.; Lee, M.; Simpson, J.M.; Hansen, R. Upper Limb Progressive Resistance Training and Stretching Exercises Following Surgery for Early Breast Cancer: A Randomized Controlled Trial. Breast Cancer Res. Treat. 2012, 133, 667–676.
66. Schmidt, T.; Berner, J.; Jonat, W.; Weisser, B.; Röcken, C.; Van Mackelenbergh, M.; et al. Influence of Arm Crank Ergometry on Development of Lymphoedema in Breast Cancer Patients after Axillary Dissection: A Randomized Controlled Trial. J. Rehabil. Med. 2017, 49, 78–83.
67. Schmitz, K.H.; Ahmed, R.L.; Troxel, A.B.; Cheville, A.; Lewis-Grant, L.; Smith, R.; Bryan, C.J.; Williams-Smith, C.T.; Chittams, J. Weight Lifting for Women at Risk for Breast Cancer-Related Lymphedema: A Randomized Trial. JAMA 2010, 304, 2699–2705

Supplement S6: Risk of bias in the included studies

Preliminary outcomes:

Comparison 1. Resistance training (RT) versus activity restriction

a) Occurrence of lymphedema (> 6 weeks):

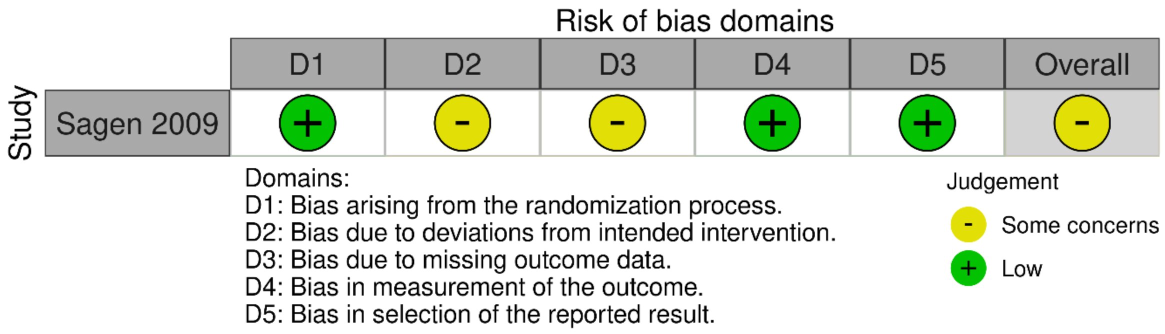

b) Arm volume (> 6 weeks):

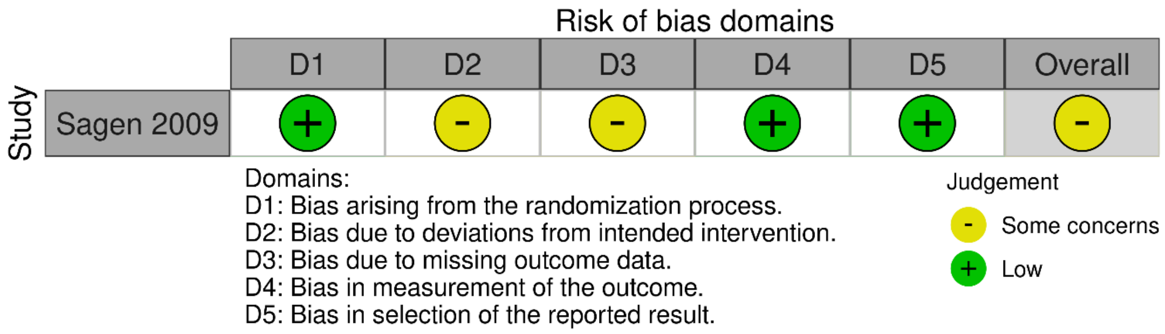

c) Pain (>6 weeks):

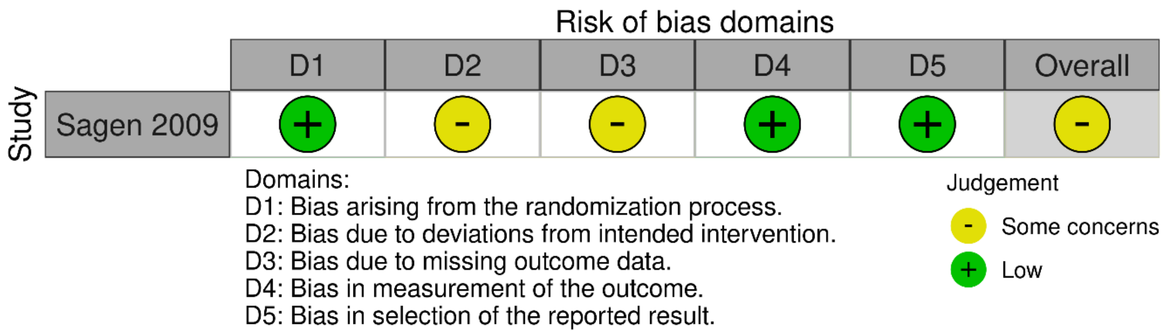

d) Adverse events:

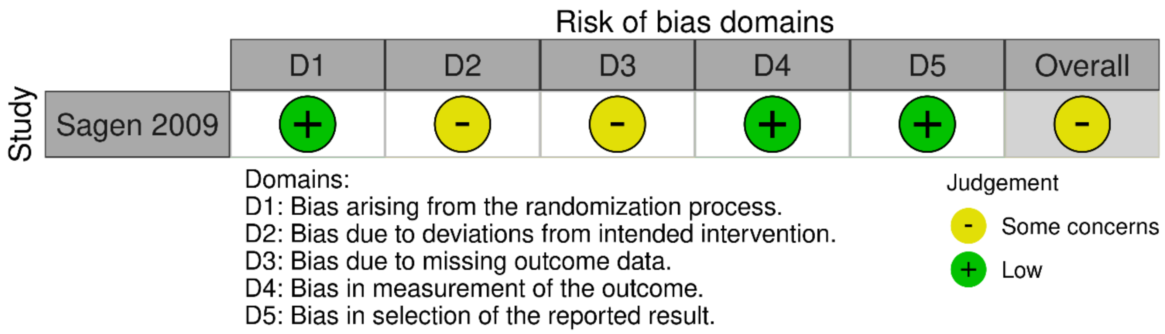

Comparison 2. Resistance training (RT) versus usual care / no structured exercise

Primary outcomes:

a) Occurrence of lymphedema (> 3 weeks to 6 weeks):

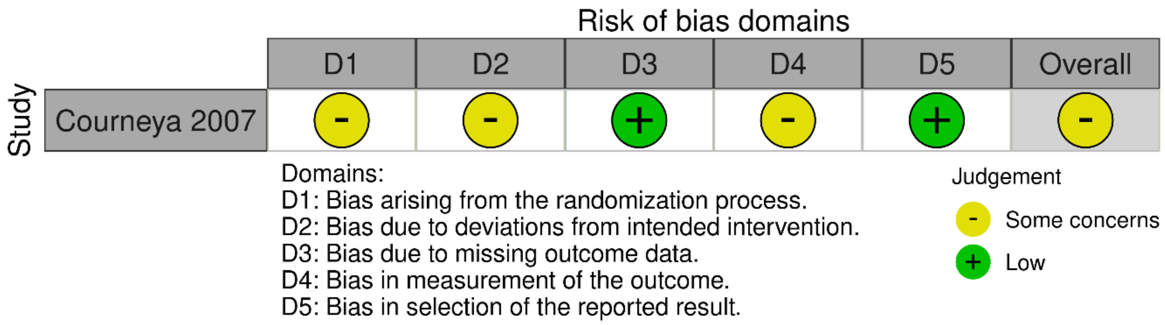

b) Occurrence of lymphedema (> 6 weeks):

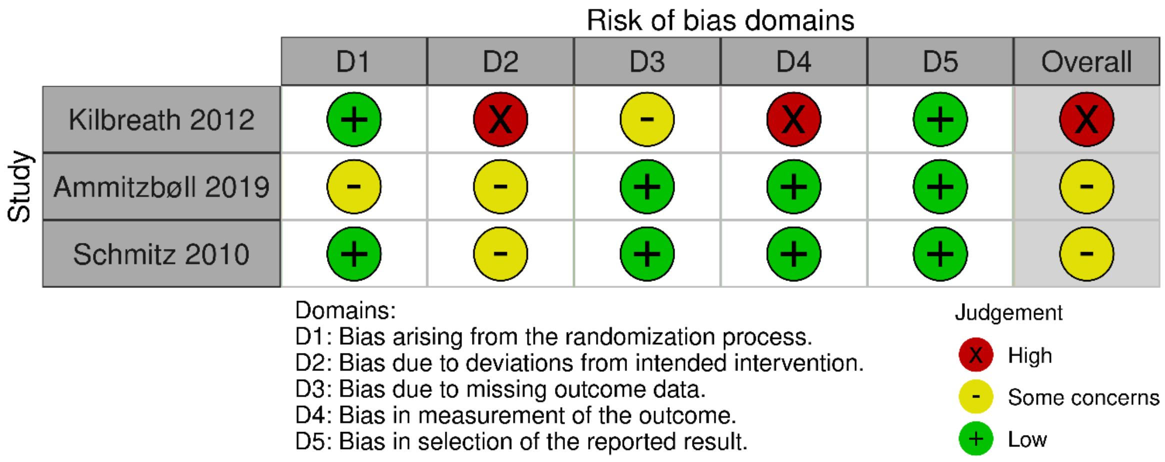

c) Arm volume (> 6 weeks):

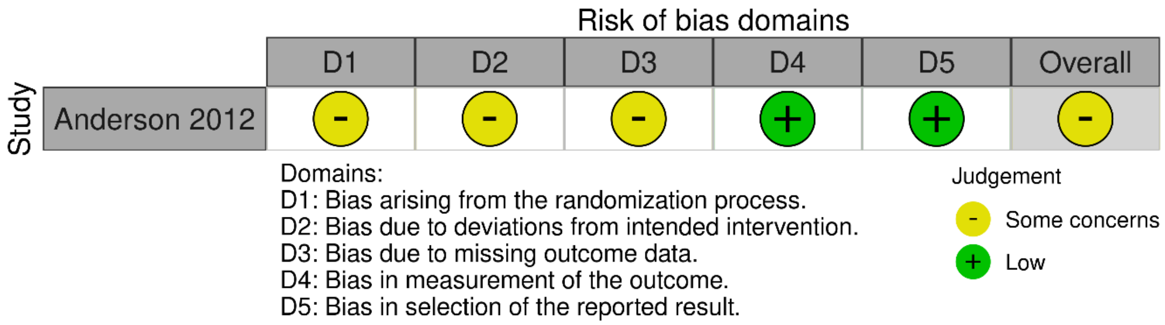

d) Overall quality of life score (> 6 weeks):

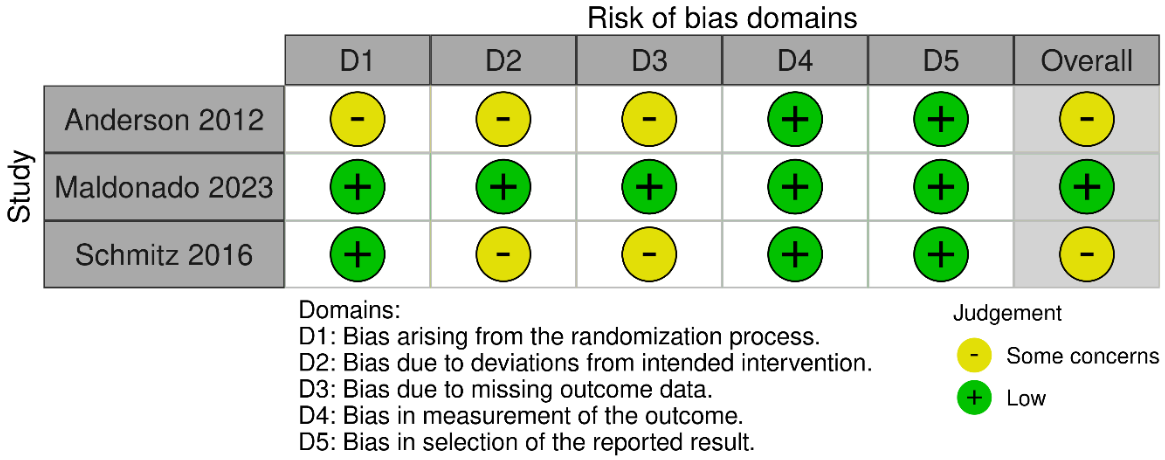

Secondary outcomes:

e) Range of motion – Shoulder flexion (> 6 weeks):

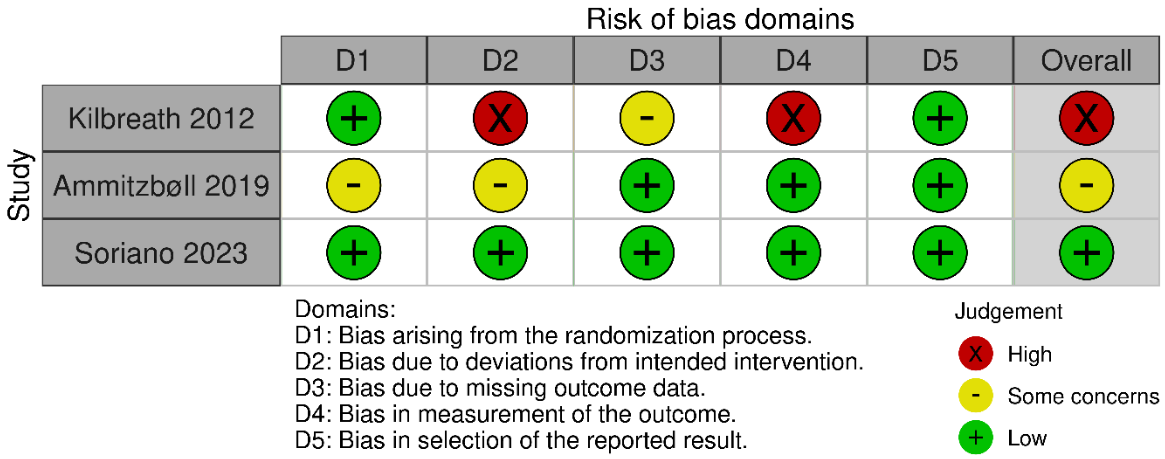

f) Range of motion – Shoulder abduction (> 6 weeks):

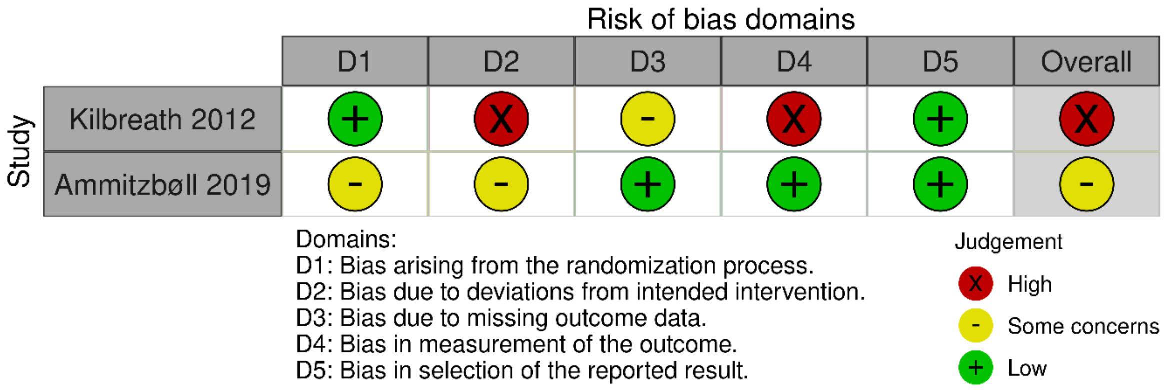

g) Range of motion – External shoulder rotation (> 6 weeks):

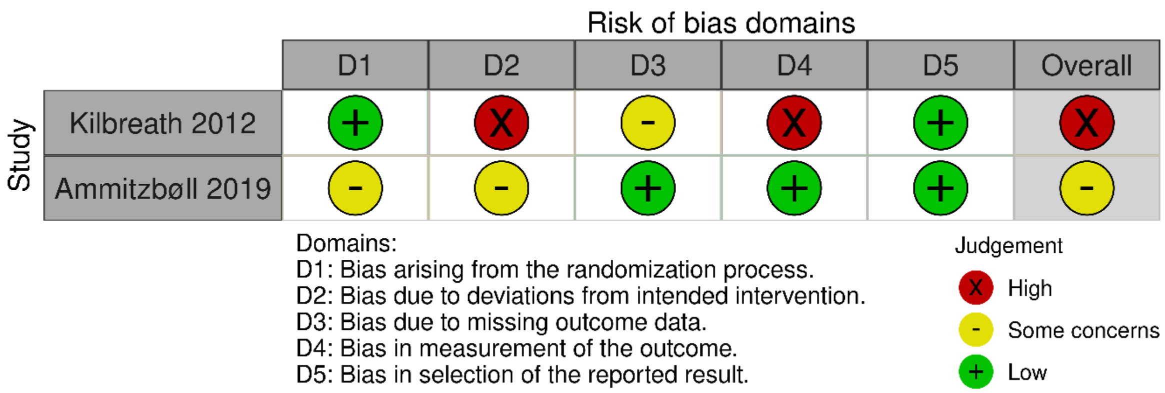

a) Grip strength (> 6 weeks):

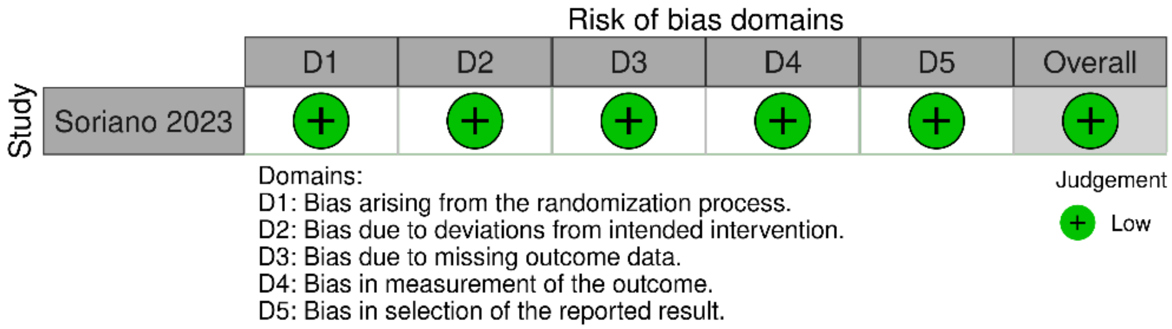

b) Pain intensity (> 6 weeks):

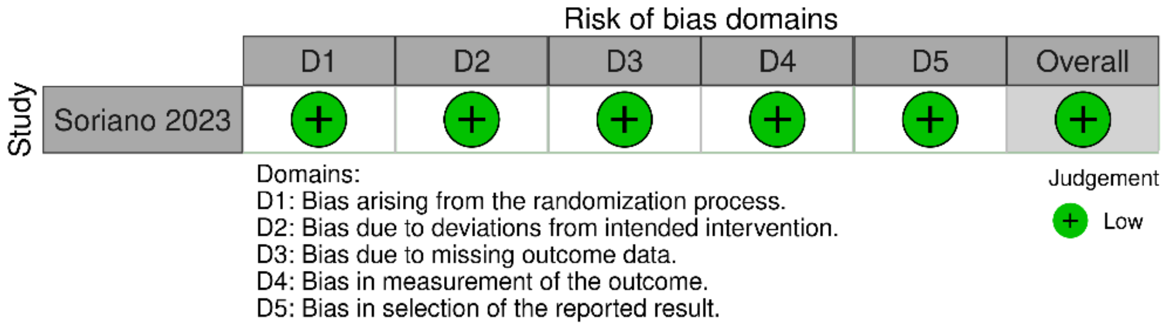

c) Adverse events:

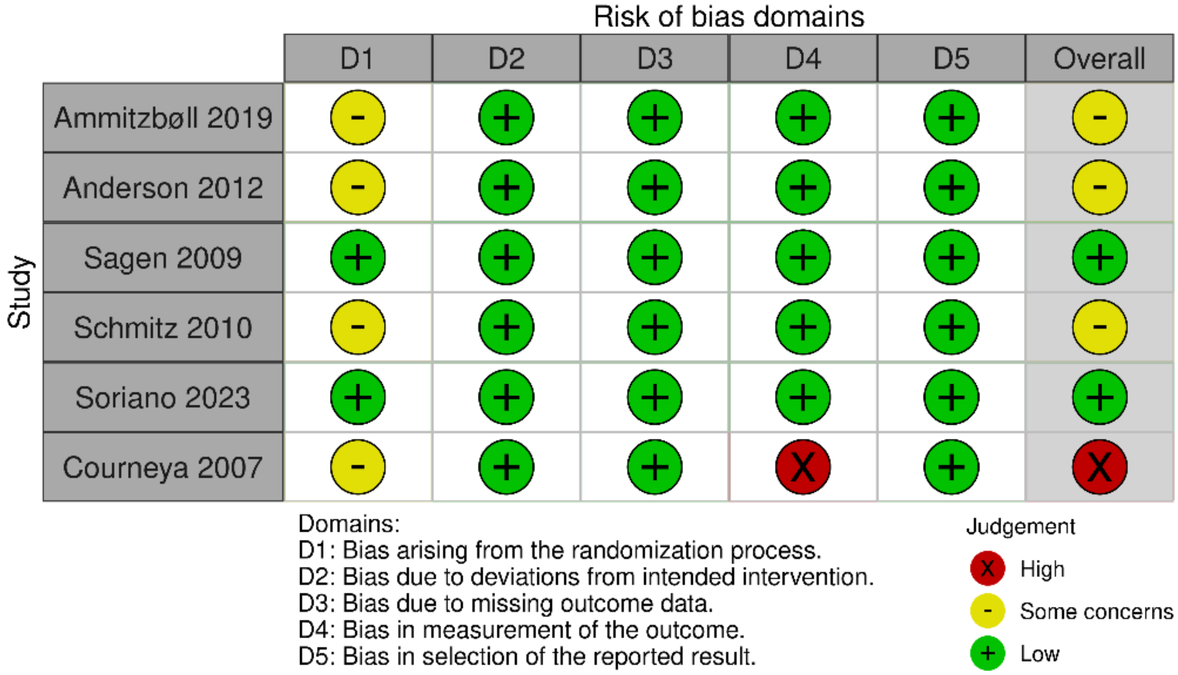

Comparator 3. RT vs aerobic training

a) Occurrence of lymphedema (> 3 weeks to 6 weeks):

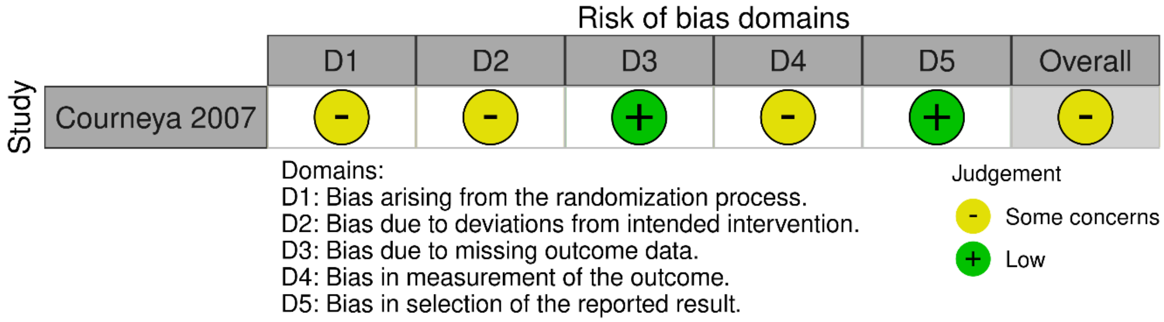

b) Adverse events:

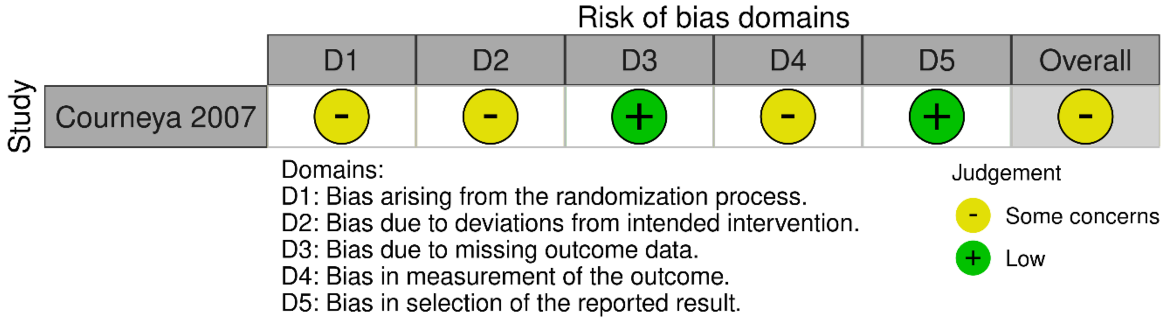

Supplement S7: Summary of findings (SoF) table for comparison 1: resistance training vs. activity restriction

Resistance training (RT) compared with activity restriction for Occurrence of lymphedema

Certainty assessment							Summary of findings				
Participants (studies) follow-up	Risk of bias	Inconsistency	Indirectness	Imprecision	Publication bias	Overall certainty of evidence	Study event rates (%)		Relative effect (95% CI)	Anticipated absolute effects	
							With activity restriction	With Resistance training (RT)		Risk with activity restriction	Risk difference with Resistance training (RT)
**Occurrence of lymphedema (follow-up: median 6 weeks)**											
204 (1 RCT)	serious[a]	not serious	not serious	very serious [b]	none	⊕◯◯◯ Very low[a,b]	13/100 (13.0%)	14/104 (13.5%)	RR 1.04 (0.51 to 2.09)	13/100 (13.0%)	5 more per 1000 (from 64 fewer to 142 more)
**Arm volume (follow-up: median 6 weeks)**											
204 (1 RCT)	serious[c]	not serious	not serious	very serious [d]	none	⊕◯◯◯ Very low[c,d]	100	104	-	100	MD 30 lower (73.64 lower to 13.64 higher)
**Pain**											
204 (1 RCT)	serious[e]	not serious	not serious	very serious [f]	none	⊕◯◯◯ Very low[e,f]	An RCT with 204 participants (*Sagen 2009*) indicated that resistance training could increase pain in 6 months, although no differences were observed in 2 years. No numerical data were reported, so the analysis was descriptive. The certainty of the evidence was very low, with uncertainty about the effect of resistance training on pain.				

CI: Confidence interval; MD: Mean difference; RR: Risk ratio; RCT: Randomized controlled trial

Explanations

a. Only study with moderate risk of bias: two domains with “some concerns” (deviations from the intervention and missing data). Considerable loss to follow-up (~25%), especially in one group, which could have affected the outcome.

b. Downgraded by two levels for very serious imprecision because the confidence interval for the effect includes both clinically important benefit and harm. This prevents confidence that the true effect of resistance training on lymphedema is null or non-important.

c. Only study with moderate risk of bias: two domains with “some concerns” (intervention deviations and missing data). Considerable loss to follow-up (~25%), especially in one group, which could have affected the outcome.

d. The confidence interval includes both a clinically important reduction and an increase in arm volume, creating significant uncertainty about whether the treatment effect is beneficial, harmful, or null. The certainty was downgraded by two levels due to very serious imprecision.

e. The study presented “some concerns” regarding deviations from the protocol and missing data. Given that the outcome (pain) is subjective, these limitations could have affected the validity of the result. One level was downgraded due to risk of bias. Only study with moderate risk of bias: two domains with “some concerns” (deviations from the intervention and missing data). Considerable loss to follow-up (~25%), especially in one group, which could have affected the result.

f. No numerical outcome data were reported, so it was not possible to estimate the effect size or precision. This uncertainty justifies downgrading by two levels due to very serious imprecision.

Supplement S8. Summary of findings (SoF) table for comparison 2: resistance training vs. usual care/no structured exercise

Resistance training (RT) compared with versus usual care / no structured exercise for women at risk of lymphedema

Certainty assessment							Summary of findings				
							Study event rates (%)		Relative effect (95% CI)	Anticipated absolute effects	
Participants (studies) follow-up	Risk of bias	Inconsistency	Indirectness	Imprecision	Publication bias	Overall certainty of evidence	With usual care / no structured exercise	With Resistance training (RT)		Risk with usual care /no structured exercise	The difference in risk with Resistance training (RT)
**Occurrence of lymphedema (> 3 weeks to 6 weeks) (follow-up: range 3 weeks to 6 weeks)**											
164 (1 RCT)	serious[a]	not serious	not serious	very serious [b]	none	⊕◯◯◯ Very low[a,b]	3/82 (3.7%)	6/82 (7.3%)	**RR 0.50** (0.13 to 1.93)	3/82 (3.7%)	**18 fewer per 1000** (from 32 fewer to 34 more)
**Occurrence of lymphedema (> 6 weeks): (follow-up: mean 6 weeks)**											
452 (3 RCTs)	serious[c]	not serious	not serious	very serious [d]	none	⊕◯◯◯ Very low[c,d]	45/223 (20.2%)	47/229 (20.5%)	**RR 0.92** (0.53 to 1.61)	45/223 (20.2%)	**16 fewer per 1000** (from 95 fewer to 123 more)
**Arm volume (follow-up: mean 6 weeks)**											
82 (1 RCT)	serious[e]	not serious	not serious	very serious [f]	none	⊕◯◯◯ Very low[e,f]	The intervention group showed a smaller increase in arm volume (+27.3 mL) compared to the control group (+57.4 mL) at 18 months, but the difference was not statistically significant (p = 0.535).				

Resistance training (RT) compared with versus usual care / no structured exercise for women at risk of lymphedema

Certainty assessment							Summary of findings				
**Overall quality of life (follow-up: mean 6 weeks)**											
131<br>(2 RCTs)	serious[g]	not serious	not serious	very serious [h]	none	⊕◯◯◯<br>Very low[g,h]	71	60	-	-	SMD **0.25 SD higher.**<br>(0.6 lower to 1.09 higher.)
**Overall quality of life (follow-up: mean 6 weeks)**											
60<br>(1 RCT)	not serious	not serious	not serious	serious[i]	none	⊕⊕⊕◯<br>Moderate[i]	A randomized clinical trial (n = 60) compared supervised resistance training plus home-based physical activity with home-based activity alone. No significant differences in overall quality of life were observed (MD: −2.9 points; CI 95 %: −7.0 to 2.1). The certainty of the evidence was rated as moderate due to imprecision.				

CI: Confidence interval; **RR:** Risk ratio; **SMD:** Standardized mean difference; RCT: Randomized controlled trial.

Explanations

a. One level was downgraded for risk of bias due to concerns about the randomization process and allocation concealment. Although the outcome was measured objectively, problems with randomization could have introduced prognostic imbalances between groups.

b. Two levels were downgraded for imprecision, as the confidence interval includes beneficial, null, and harmful effects.

c. One level was downgraded for risk of bias: one study had a high risk of intervention bias (D2), which could have influenced the results. Although the outcome was measured objectively, this limitation remains relevant.

d. Two levels were downgraded for imprecision: the confidence interval crosses relevant thresholds, including important benefit, no effect, and possible harm.

e. One level was downgraded for risk of bias due to concerns about randomization and missing data. Although the outcome was measured objectively, these limitations could have affected the validity of the results.

f. Two levels were downgraded due to imprecision: the sample size was small, the confidence interval was wide, and statistical significance was not achieved. It was not determined whether the observed difference (~30 mL) is clinically important.

g. One level was downgraded due to risk of bias due to methodological concerns.

h. Two levels were downgraded due to serious imprecision: the confidence interval is wide and crosses multiple clinically relevant thresholds (including no effect, benefit, and harm).

i. One level was downgraded due to imprecision: the confidence interval is wide and includes both no effect and possible clinically insignificant improvements or deteriorations. The study had a low risk of bias.

Resistance training (RT) compared with versus usual care / no structured exercise for women at risk of lymphedema

Certainty assessment							Summary of findings				
							Study event rates (%)		Relative effect (95% CI)	Anticipated absolute effects	
Participants (studies) follow-up	Risk of bias	Inconsistency	Indirectness	Imprecision	Publication bias	Overall certainty of evidence	With usual care /no structured exercise	With Resistance training (RT)		Risk with versus usual care /no structured exercise	The difference in risk with Resistance training (RT)
**ROM: Shoulder flexion (follow-up: mean 6 weeks; assessed with: Goniometer)**											
130 (1 RCT)	serious[a]	not serious	not serious	serious[b]	none	⊕⊕◯◯ Low[a,b]	62	68	-	62	MD **1 Degrees lower** (5.64 lower than 3.64 higher.)
**ROM: Shoulder flexion (follow-up: mean 6 weeks; assessed with: digital inclinometer)**											
141 (1 RCT)	very serious [c]	not serious	not serious	serious[d]	none	⊕◯◯◯ Very low[c,d]	One RCT (n = 141) compared a supervised exercise program with education on shoulder flexion change at more than 6 months. The difference between groups was small (MD: 1.9°, CI 95 %: −4.5 to 8.2), with no statistical significance. The certainty of the evidence was rated as very low due to high risk of bias and imprecision.				
**ROM: Shoulder flexion (assessed with: Electrogoniometer)**											
60 (1 RCT)	not serious	not serious	not serious	very serious [e]	none	⊕⊕◯◯ Low[e]	One RCT (n = 60) assessed the effect of a supervised resistance training program on change in shoulder flexion at 12 weeks. No statistically significant differences were observed between groups (MD: −2.0 degrees; CI 95 %: −8.3 to 4.4). The certainty of the evidence was **low** due to very serious imprecision.				
**Shoulder abduction (follow-up: mean 6 weeks; assessed with: digital inclinometer)**											

Resistance training (RT) compared with versus usual care / no structured exercise for women at risk of lymphedema

Certainty assessment							Summary of findings				
130<br>(1 RCT)	serious[f]	not serious	not serious	very serious [g]	none	⊕◯◯◯<br>Very low[f,g]	62	68	-	62	MD 2 Degrees lower<br>(11.86 lower than 7.86 higher.)
**Shoulder abduction (follow-up: mean 6 weeks; assessed with: digital inclinometer)**											
141<br>(1 RCT)	very serious [h]	not serious	not serious	serious[i]	none	⊕◯◯◯<br>Very low[h,i]	A randomized clinical trial (n = 141) evaluated the effect of an 8-week supervised resistance training program on shoulder abduction in women who had undergone breast cancer surgery. At 6 months, the exercise group showed a greater increase in abduction (mean: 20.1°) compared to the control group (mean: 10.1°), with a mean difference of 10.0° (CI 95 %: 3.6 to 16.5). Due to the high risk of bias from deviations in the intervention and outcome measurement, the study is reported narratively, and the certainty of evidence was rated as very low.				
**External shoulder rotation (follow-up: mean 6 weeks; assessed with: Goniometer)**											
130<br>(1 RCT)	serious[j]	not serious	not serious	very serious [k]	none	⊕◯◯◯<br>Very low[j,k]	62	68	-	62	MD 1 Degree higher.<br>(5.02 lower than 7.02 higher.)
**External shoulder rotation (follow-up: mean 6 weeks; assessed with: Goniometer)**											
151<br>(1 RCT)	very serious [l]	not serious	not serious	serious[m]	none	⊕◯◯◯<br>Very low[l,m]	One RCT (n ≈ 151) compared an exercise program with education on shoulder external rotation at >6 months. The difference between groups was small and not significant (MD −1.2°; CI 95% −6.2 to 3.8). The certainty of evidence was rated as very low due to high risk of bias and imprecision.				

Resistance training (RT) compared with versus usual care / no structured exercise for women at risk of lymphedema

Certainty assessment							Summary of findings
**Pain (follow-up: mean 6 weeks; assessed with: Brief Pain Inventory – Short Form (BPI-SF))**							
60 (1 RCT)	not serious	not serious	not serious	serious[n]	none	⊕⊕⊕◯ Moderate[n]	One RCT (n = 60) assessed pain intensity using the Brief Pain Inventory – Short Form (BPI-SF) after 12 weeks of supervised resistance training plus home-based physical activity versus control (home-based activity alone). No significant differences were observed between groups; the distribution of pain scores was similar. The certainty of the evidence was rated as moderate due to imprecision.
**Grip strength (follow-up: mean 6 weeks; assessed with: electromechanical dynamometer)**							
60 (1 RCT)	not serious	not serious	not serious	very serious [o]	none	⊕⊕◯◯ Low[o]	A supervised 12-week resistance training program produced a small increase in handgrip strength compared with home-based physical activity alone, but the effect was uncertain. The mean difference between groups was 0.2 kg (CI 95%: −1.3 to 1.6), which includes both no effect and possible benefits or even slight harm.

CI: Confidence interval; MD: Mean difference; RR: Risk ratio; SMD: Standardized mean difference; RCT: Randomized controlled trial

Explanations

a. One level was downgraded due to risk of bias caused by concerns in the domains of randomization and deviations from the intervention.

b. An additional level was downgraded due to imprecision: the confidence interval is wide and includes both a slight decrease and a slight increase in shoulder flexion.

c. Two levels were downgraded because of risk of bias due to relevant deviations from the intervention and possible bias in outcome measurement.

d. An additional level was downgraded because of imprecision, as the difference was small and the confidence interval was not reported, but probably included no clinically relevant effect.

e. Two levels were downgraded due to serious imprecision: the confidence interval is wide and crosses relevant thresholds, including no effect, benefit, and possible worsening. The sample size was small and the estimated effect uncertain.

f. One level was downgraded due to risk of bias due to concerns about randomization and deviations of the intervention.

g. Two levels were downgraded due to very serious imprecision: the confidence interval was wide, including no effect, possible improvement, and slight deterioration.

h. Two levels were downgraded because of risk of bias due to major deviations from the intervention and possible bias in outcome measurement.

i. An additional level was downgraded because of imprecision: the confidence interval is wide and crosses clinically relevant thresholds, including no effect and small changes.

j. One level was downgraded because of risk of bias (randomization and deviations of the intervention).

k. Two levels were downgraded due to serious imprecision: the confidence interval includes no effect, slight benefit, and slight worsening, with a limited sample siz.

l. Two levels were downgraded due to risk of bias (deviations of the intervention and possible bias in the measurement of the outcome)

m. One level was downgraded due to imprecision (the CI covers no effect and covers small changes in both directions.

n. One level was downgraded due to imprecision: the sample size was small, and the distribution of results does not allow us to rule out the absence of a clinically relevant effect.

o. We downgraded two levels due to imprecision because the confidence interval (−1.3 to 1.6 kg) crosses clinically relevant thresholds for harm, benefit, and trivial effect, generating significant uncertainty about the actual effect of the intervention.

Supplement S9: Summary of findings (SoF) table for Comparison 3: resistance training vs. aerobic training

Resistance training (RT) compared with aerobic training for women at risk of lymphedema

Certainty assessment							Summary of findings				
							Study event rates (%)		Relative effect (95% CI)	Anticipated absolute effects	
Participants (studies) follow-up	Risk of bias	Inconsistency	Indirectness	Imprecision	Publication bias	Overall certainty of evidence	With aerobic training	With Resistance training (RT)		Risk with aerobic training	Difference in risk with Resistance training (RT)
**Occurrence of lymphedema (follow-up: range 3 weeks to 6 weeks)**											
160 (1 RCT)	serious[a]	not serious	not serious	very serious [b]	none	⨁◯◯◯ Very low[a,b]	7/78 (9.0%)	3/82 (3.7%)	RR 0.41 (0.11 to 1.52)	7/78 (9.0%)	53 fewer per 1000 (from 80 fewer to 47 more)

CI: Confidence interval; RR: Risk ratio; RCT: Randomized controlled trial

Explanations

a. We downgraded one level due to risk of bias because of “some concerns” in the domains of randomization, intervention deviation, and outcome measurement.

b. We downgraded two levels for very serious imprecision, as the confidence interval (RR: 0.41; CI 95%: 0.11 to 1.52) includes both a significant benefit and possible harm and is based on a very low number of events (only 10 in total), which generates high uncertainty about the actual effect.

Supplement S10. Risk of bias in sensitivity analysis according to effect measure (SMD vs. MD in overall quality of life)

a) <u>Overall quality of life score (> 6 weeks):</u>

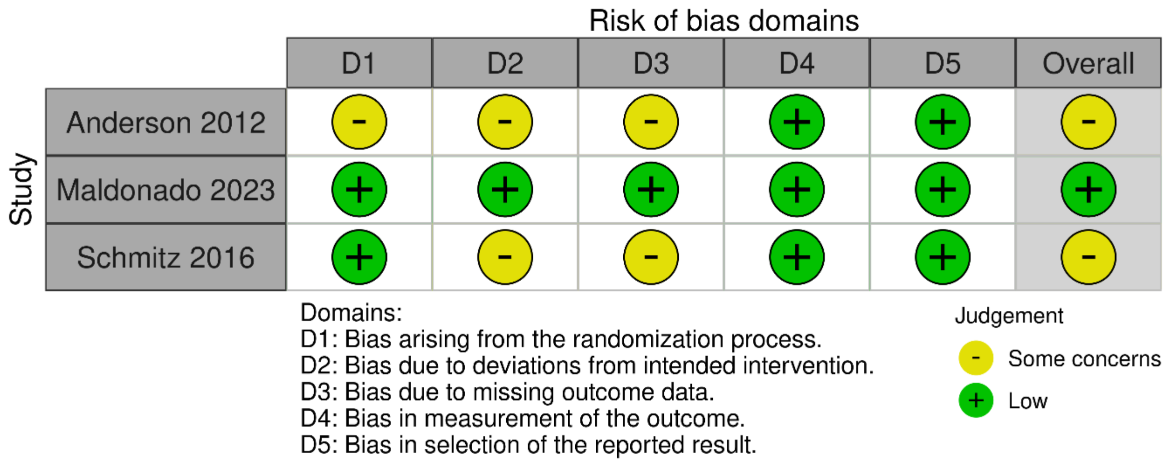

Supplement S11. Certainty of evidence assessment (GRADE) in sensitivity analysis according to effect measure (SMD vs. MD in overall quality of life)

Resistance training compared with usual care / no structured exercise for women at risk of lymphedema

Certainty assessment							Summary of findings				
							Study event rates (%)		Relative effect (95% CI)	Anticipated absolute effects	
Participants (studies) follow-up	Risk of bias	Inconsistency	Indirectness	Imprecision	Publication bias	Overall certainty of evidence	With usual care /no structured exercise	With Resistance Training		Risk with usual care /no structured exercise	Difference in risk with resistance training
**Overall quality of life (follow-up: mean 6 week)**											
131 (2 randomized controlled trials [RCTs])	serious[a]	not serious	not serious	very serious[b]	none	⊕◯◯◯ Very low[a,b]	71	60	-	71	MD **1.34 higher.** (0.28 higher to 2.39 higher.)

CI: Confidence interval; MD: Mean difference; RCT: Randomized controlled trials

Explanations

a. Both included studies present at least "some concerns" in key domains of risk of bias (particularly in domains D1 to D3, which address random sequence generation, allocation concealment, and deviations from the protocol).

b. Crosses two or more thresholds, should be downgraded by two levels for very serious imprecision.

Supplement S12. Risk of bias in sensitivity analysis according to diagnostic criteria for lymphedema

a) Studies with diagnostic threshold (≥10 % difference in volume between arms):

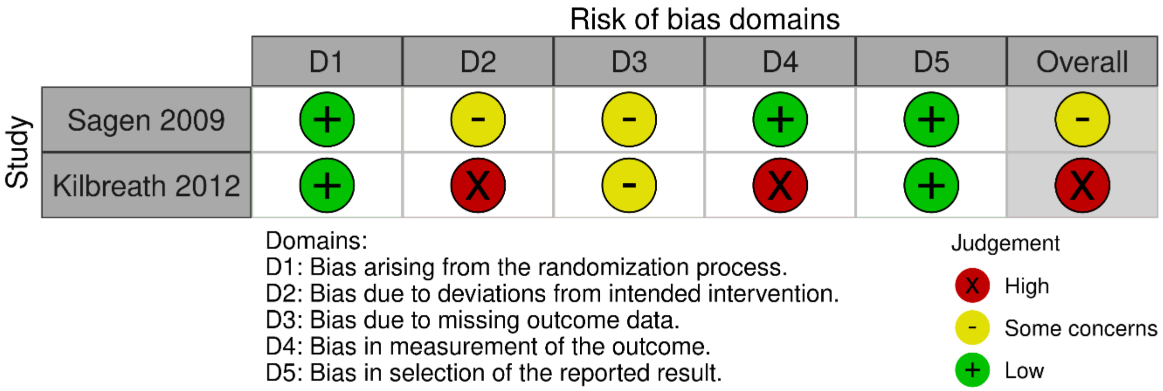

b) Studies with diagnostic thresholds (≥10 % and ≥5 %), excluding criteria (>3 %):

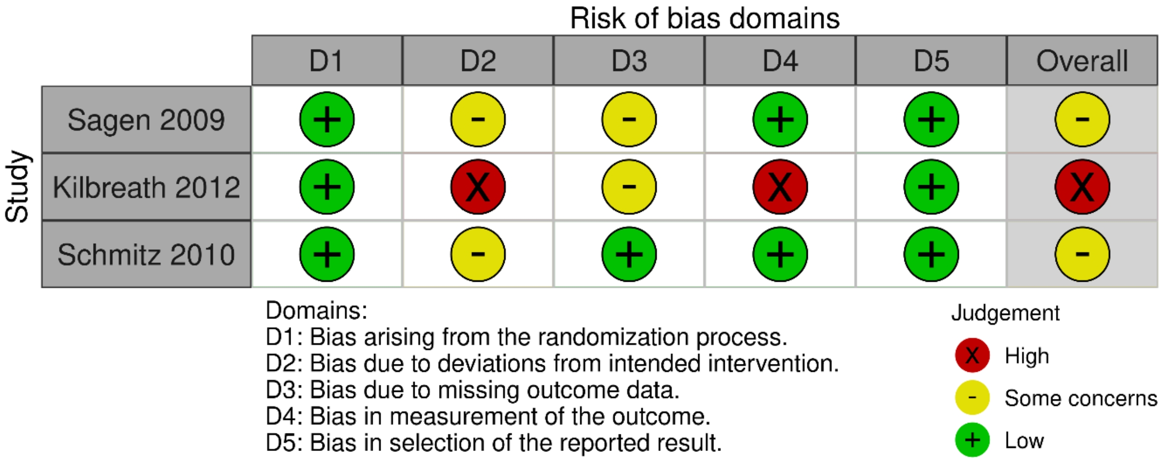

c) All studies with follow-up longer than 6 weeks, regardless of diagnostic criteria:

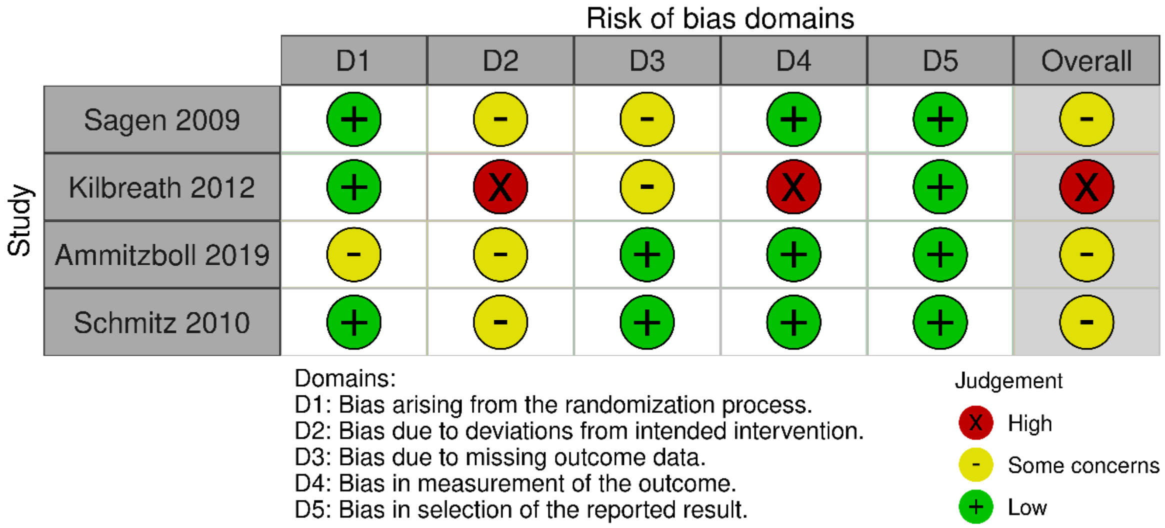
Risk of bias domains
D1
D2
D3
D4
D5
Overall
Study
Sagen 2009
Kilbreath 2012
Ammitzboll 2019
Schmitz 2010
Domains:
D1: Bias arising from the randomization process.
D2: Bias due to deviations from intended intervention.
D3: Bias due to missing outcome data.
D4: Bias in measurement of the outcome.
D5: Bias in selection of the reported result.
Judgement
High
Some concerns
Low

Supplement S13. Certainty of evidence assessment (GRADE) in sensitivity analysis according to diagnostic criteria for lymphedema

Resistance training compared with activity restriction; usual care / no structured exercise; aerobic training for female breast cancer survivors at risk of lymphedema, defined by diagnostic thresholds of ≥5 % or ≥10 %, excluding criteria >3%

Certainty assessment							Summary of findings				
Participants (studies) follow-up	Risk of bias	Inconsistency	Indirectness	Imprecision	Publication bias	Overall certainty of evidence	Study event rates (%)		Relative effect (95% CI)	Anticipated absolute effects	
							With activity restriction;usual care / no structured exercise; aerobic training	With Resistance Training		Risk with activity restriction; usual care / no structured exercise; aerobic training	Difference in risk with resistance training
**Occurrence of lymphedema (follow-up: median 6 weeks)**											
498 (3 randomized controlled trials [RCTs])	serious [a]	not serious	not serious	very serious [b]	none	⊕◯◯◯ Very low[a,b]	35/247 (14.2%)	31/251 (12.4%)	**RR 0.79** (0.50 to 1.27)	35/247 (14.2%)	**30 less per 1000** (from 71 less to 38 more)

CI: Confidence interval; **RR:** Risk ratio; RCT: Randomized controlled trials

Explanations

a. One of the included studies presented a high risk of bias in critical domains, without sensitivity analyses to discard its impact.

b. The confidence interval crosses two clinically relevant thresholds, ranging from significant benefit to possible harm, indicating very serious imprecision.

Resistance training compared with activity restriction; usual care / no structured exercise; aerobic training for female breast cancer survivors at risk of lymphedema (defined as interarm volume difference ≥10%)

Certainty assessment							Summary of findings				
							Study event rates (%)			Anticipated absolute effects	
Participants (studies) follow-up	Risk of bias	Inconsistency	Indirectness	Imprecision	Publication bias	Overall certainty of evidence	With activity restriction; usual care / no structured exercise; aerobic training	With Resistance Training	Relative effect (95% CI)	Risk with activity restriction; usual care / no structured exercise; aerobic training	Difference in risk with resistance training
**Occurrence of lymphedema (follow-up: median 6 weeks)**											
351 (2 randomized controlled trials [RCTs])	serious [a]	not serious	not serious	very serious [b]	none	⊕◯◯◯ Very low[a,b]	22/172 (12.8%)	20/179 (11.2%)	**RR 0.88** (0.50 to 1.56)	22/172 (12.8%)	**15 less per 1000** (from 64 less to 72 more)

CI: Confidence interval; **RR:** Risk ratio; RCT: Randomized controlled trials.

Explanations

a. A study with a high risk of bias in critical domains (protocol deviation and outcome measurement) without sensitivity analysis to discard its impact. The number of studies is low, which increases the influence of this bias on the overall estimate.

b. The 95% CI of the estimate crosses two clinically important thresholds (from moderate benefit to significant harm). Low sample size.

Resistance training compared with activity restriction, usual care, no structured exercise, or aerobic training for female breast cancer survivors at risk of lymphedema, defined by all diagnostic criteria used in the included studies.

Certainty assessment							Summary of findings				
							Study event rates (%)			Anticipated absolute effects	
Participants (studies) follow-up	Risk of bias	Inconsistency	Indirectness	Imprecision	Publication bias	Overall certainty of evidence	With activity restriction, usual care, no structured exercise or aerobic training	With Resistance Training	Relative effect (95% CI)	Risk with activity restriction, usual care, no structured exercise or aerobic training	Difference in risk with resistance training
**Ocurrencia de linfedema**											
656 (4 randomized controlled trials [RCTs])	serious [a]	not serious	not serious	very serious [b]	none	⊕◯◯◯ Very low[a,b]	58/323 (18.0%)	61/333 (18.3%)	**RR 0.99** (0.67 to 1.46)	58/323 (18.0%)	**2 less per 1000** (from 59 less to 83 more)

CI: Confidence interval; **RR:** Risk ratio; RCT: Randomized controlled trials.

Explanations

a. One level was downgraded due to the presence of a study with a high risk of bias in critical domains, without sensitivity analysis to discard its impact on the results.

b. The confidence interval crosses two clinically relevant thresholds, ranging from significant benefit to possible harm, indicating very serious imprecision.

Supplement S14. Risk of bias in sensitivity analysis excluding studies at high risk of bias

Comparison 2. Resistance training (RT) versus usual care / no structured exercise

a) <u>Occurrence of lymphedema (> 6 weeks):</u>

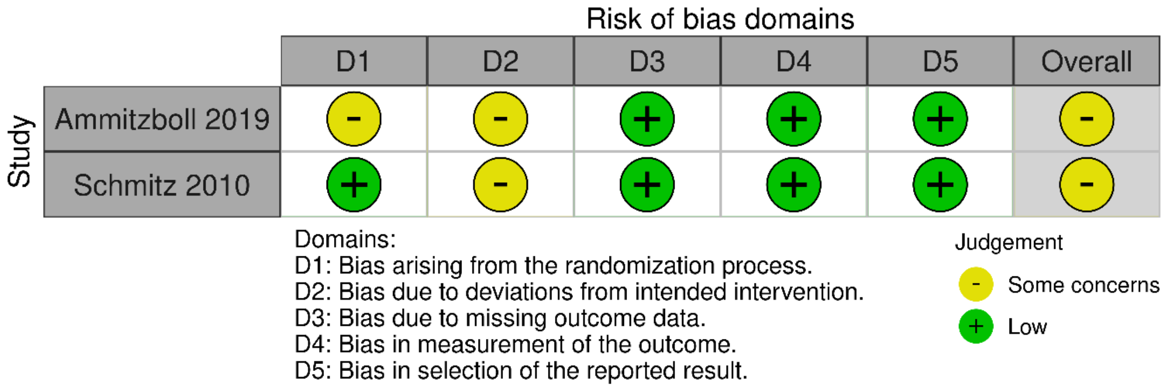

Secondary outcomes:

b) <u>Range of motion – Shoulder flexion (> 6 weeks):</u>

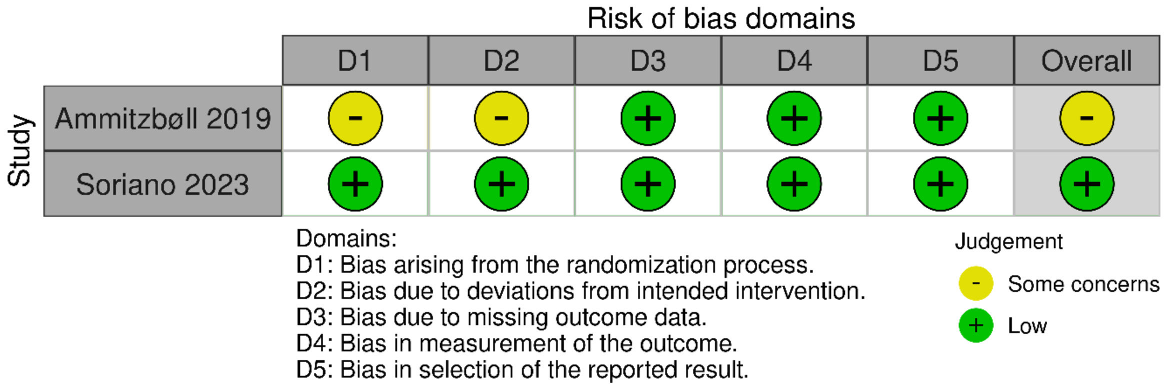

c) <u>Range of motion – Shoulder abduction (> 6 weeks):</u>

Risk of bias domains

Study	D1	D2	D3	D4	D5	Overall
Ammitzbøll 2019	-	-	+	+	+	-

Domains:
D1: Bias arising from the randomization process.
D2: Bias due to deviations from intended intervention.
D3: Bias due to missing outcome data.
D4: Bias in measurement of the outcome.
D5: Bias in selection of the reported result.

Judgement
- Some concerns
+ Low

d) <u>External shoulder rotation (> 6 weeks):</u>

Risk of bias domains

Study	D1	D2	D3	D4	D5	Overall
Ammitzbøll 2019	-	-	+	+	+	-

Domains:
D1: Bias arising from the randomization process.
D2: Bias due to deviations from intended intervention.
D3: Bias due to missing outcome data.
D4: Bias in measurement of the outcome.
D5: Bias in selection of the reported result.

Judgement
- Some concerns
+ Low

e) <u>Adverse events:</u>

Risk of bias domains

Study	D1	D2	D3	D4	D5	Overall
Sagen 2009	+	-	-	+	+	-
Ammitzboll 2019	-	-	+	+	+	-
Schmitz 2010	+	-	+	+	+	-
Anderson 2012	-	-	-	+	+	-
Maldonado-Soriano 2023	+	+	+	+	+	+

Domains:
D1: Bias arising from the randomization process.
D2: Bias due to deviations from intended intervention.
D3: Bias due to missing outcome data.
D4: Bias in measurement of the outcome.
D5: Bias in selection of the reported result.

Judgement
- Some concerns
+ Low

Supplement S15. Certainty of evidence assessment (GRADE) in sensitivity analysis excluding studies at high risk of bias

Resistance training (RT) compared with usual care / no structured exercise for risk of lymphedema

Certainty assessment							Summary of findings				
Participants (studies) follow-up	Risk of bias	Inconsistency	Indirectness	Imprecision	Publication bias	Overall certainty of evidence	Study event rates (%)		Relative effect (95% CI)	Anticipated absolute effects	
							With usual care / no structured exercise	With Resistance training (RT)		Risk with usual care / no structured exercise	Difference in risk with Resistance training (RT)
**Occurrence of lymphedema (follow-up: median 6 weeks)**											
305 (2 RCTs)	not serious	not serious	not serious	very serious [a]	none	⊕⊕◯◯ Low[a]	36/151 (23.8%)	41/154 (26.6%)	**RR 1.01** (0.50 to 2.02)	36/151 (23.8%)	**2 more per 1000** (from 119 fewer to 243 more)
**Shoulder flexion (follow-up: mean 6 weeks)**											
130 (1 RCT)	not serious	not serious	not serious	very serious [b]	none	⊕⊕◯◯ Low[b]	62	68	-	62	MD **1 Degrees lower** (5.64 lower than 3.64 higher.)

Shoulder flexion (follow-up: mean 6 weeks)

Resistance training (RT) compared with usual care / no structured exercise for risk of lymphedema

Certainty assessment							Summary of findings				
60<br>(1 RCT)	not serious	not serious	not serious	very serious [c]	none	⊕⊕◯◯<br>Low[c]	A randomized study (n = 60) compared supervised resistance training with no exercise and found no conclusive evidence of benefit or harm in the range of motion (ROM) of shoulder flexion in the affected arm. The mean difference in change was −2.0 degrees (CI 95%: −8.3 to 4.4), which includes both clinically important improvement and worsening. The certainty of the evidence was rated as low due to very serious imprecision.				
**Shoulder abduction (follow-up: mean 6 weeks)**											
130<br>(1 RCT)	serious[d]	not serious	not serious	very serious [e]	none	⊕◯◯◯<br>Very low[d,e]	62	68	-	62	MD **2 Degrees lower** (11.86 lower than 7.86 higher.)
**External shoulder rotation (follow-up: mean 6 weeks)**											
130<br>(1 RCT)	serious[f]	not serious	not serious	very serious [g]	none	⊕◯◯◯<br>Very low[f,g]	62	68	-	62	MD **1 Higher degrees.** (5.02 lower than 7.02 higher.)

CI: Confidence interval; MD: Mean difference; RR: Risk ratio; RCTs: Randomized controlled trials.

Explanations

a. Two levels were downgraded due to very serious imprecision: the CI of the RR (1.01, 0.50 to 2.02) crosses thresholds of benefit, harm, and trivial effect, indicating high uncertainty about the effect. The sample size (305) does not reduce this imprecision.

b. Two levels were downgraded due to very serious imprecision: the CI of the MD (−5.64 to 3.64) crosses thresholds of benefit, harm, and trivial effect, indicating high uncertainty about the effect.

c. Two levels were downgraded due to very serious imprecision: the CI of the MD (−8.3 to 4.4) crosses thresholds of benefit, harm, and trivial effect, generating high uncertainty about the effect.

d. One level was downgraded due to risk of bias due to lack of clarity in randomization and allocation concealment, which compromises the initial comparability between groups in the only available study.

e. Two levels were downgraded due to very serious imprecision: the CI for the mean difference (−11.86 to 7.86) crosses thresholds for benefit, harm, and trivial effect, indicating high uncertainty about the effect.

f. One level was downgraded due to risk of bias due to lack of clarity in randomization and allocation concealment, which compromises comparability between groups in the only available study.

g. Two levels were downgraded due to very serious imprecision, as the CI (−5.02 to 7.02) crosses thresholds of benefit, harm, and trivial effect.
